# Ultrastructural localization of *Porphyromonas gingivalis* gingipains in the substantia nigra of Parkinson’s disease brains

**DOI:** 10.1038/s41531-024-00705-2

**Published:** 2024-04-25

**Authors:** Florian Ermini, Victoria F. Low, Jennifer J. Song, Adelie Y. S. Tan, Richard L. M. Faull, Michael Dragunow, Maurice A. Curtis, Stephen S. Dominy

**Affiliations:** 1 Previously Cortexyme, Inc., South San Francisco, CA USA; 2 Department of Bioengineering, Stanford University, Stanford, CA USA; 3 NeuroValida, The University of Auckland, Auckland, New Zealand; 4 Department of Anatomy and Medical Imaging, The University of Auckland, Auckland, New Zealand; 5 Department of Pharmacology and Clinical Pharmacology, The University of Auckland, Auckland, New Zealand; 6 Lighthouse Pharmaceuticals, Inc., San Francisco, CA USA

**Keywords:** Parkinson's disease, Cellular neuroscience, Parkinson's disease

## Abstract

Gingipains are protease virulence factors produced by *Porphyromonas gingivalis*, a Gram-negative bacterium best known for its role in chronic periodontitis. Gingipains were recently identified in the middle temporal gyrus of postmortem Alzheimer’s disease (AD) brains, where gingipain load correlated with AD diagnosis and tau and ubiquitin pathology. Since AD and Parkinson’s disease (PD) share some overlapping pathologic features, including nigral pathology and Lewy bodies, the current study explored whether gingipains are present in the substantia nigra pars compacta of PD brains. In immunohistochemical techniques and multi-channel fluorescence studies, gingipain antigens were abundant in dopaminergic neurons in the substantia nigra of both PD and neurologically normal control brains. 3-dimensional reconstructions of Lewy body containing neurons revealed that gingipains associated with the periphery of alpha-synuclein aggregates but were occasionally observed inside aggregates. In vitro proteomic analysis demonstrated that recombinant alpha-synuclein is cleaved by lysine-gingipain, generating multiple alpha-synuclein fragments including the non-amyloid component fragments. Immunogold electron microscopy with co-labeling of gingipains and alpha-synuclein confirmed the occasional colocalization of gingipains with phosphorylated (pSER129) alpha-synuclein. In dopaminergic neurons, gingipains localized to the perinuclear cytoplasm, neuromelanin, mitochondria, and nucleus. These data suggest that gingipains localize in dopaminergic neurons in the substantia nigra and interact with alpha-synuclein.

## Introduction

The recent identification of neuronal alpha-synuclein (αSyn) as a critical antimicrobial protein involved in coordinating immune responses to a pathogenic challenge has furthered interest in identifying possible infectious causes of Parkinson’s disease (PD)^1^. PD is characterized by the loss of dopamine-containing neurons in the substantia nigra pars compacta (SNpc), resulting in motor symptoms including resting tremor, shuffling gate, and mask-like facial expression^2–4^. In surviving dopaminergic neurons of the SNpc, aggregated αSyn in perikarya and neurites of neurons can be found in the form of Lewy bodies and Lewy neurites, respectively^5^. Because of the emerging role of αSyn in immune defense, it has been suggested that pathologic αSyn accumulation in the SNpc may be due to chronic stimulation from repeated infections or loss of control of commensal bacteria in the periphery^1^. To date, multiple infectious agents have been proposed to be involved with PD^6^.

*Porphyromonas gingivalis* has emerged as a candidate bacterium in PD pathogenesis. Oral administration of *P. gingivalis* was recently shown to reduce dopaminergic neurons in the SNpc of mice carrying the leucine-rich repeat kinase 2 (*LRRK2*) R144G mutation^7^, a mutation associated with late-onset PD^8^. An increase in the level of αSyn was detected in the myenteric neurons of the colon of the *LRRK2* R144G mice given oral *P. gingivalis*^7^, consistent with evidence that αSyn is an innate immune response protein in peripheral tissues such as the gut^1,6^. *P. gingivalis* is best known for its role as a keystone pathogen in the development of chronic periodontitis (CP), one of the most prevalent and chronic peripheral infections globally^9,10^. CP and tooth loss have been shown to be associated with an increased risk of developing PD^11,12^. Several population based studies found the risk for PD to be associated with periodontal disease or tooth loss^11–15^ and prophylactic treatment against periodontal disease reduced the risk for PD^12,16^. A cross sectional study of patients with PD found that 75% had periodontal disease and that the severity of motor impairments correlated with the severity of periodontal disease^15,17^. Patients with PD suffer from reduced upper body motor function and in advanced stages impairment in the orofacial system, which may lead to reduced dental care and development of periodontal disease^13,18^, but closer analysis of dental care or nutrition showed no difference between PD and control patients^14^ and periodontal disease was worse in patients with PD compared to patients with stroke^15^. Thus, the incidence of periodontal disease and *P. gingivalis* infection in patients with PD is much higher compared to healthy subjects. The resulting chronic infection of peripheral tissues and bacterial infiltration of the central nervous system may advance the progression of PD.

A recent study identified a gingipain virulence factor produced by *P. gingivalis*, arginine-gingipain A (RgpA), in the peripheral circulation of PD patients, and experiments linked the gingipain to abnormal blood clotting in PD, prompting the authors to suggest that *P. gingivalis* and its gingipain virulence factors may be involved in the pathogenesis of PD^19^. Gingipains are cysteine proteases and are considered the major virulence factors of *P. gingivalis*, a Gram-negative, asaccharolytic anaerobic bacterium that is mainly found during gingival and periodontal infections; however, it can also be found at low levels in 25% of healthy individuals with no oral disease^20,21^. Gingipains consist of lysine-gingipain (Kgp), RgpA, and arginine-gingipain B (RgpB). Kgp cleaves host proteins on the C-terminal side of lysine residues, and RgpA and RgpB cleave proteins on the C-terminal side of arginine residues. Importantly, gingipains can act at a distance from the parent bacterium by being secreted into the extracellular milieu in outer membrane vesicles (OMV), where they inactivate host defenses, acquire iron and nutrients, and destroy tissues^20,22,23^. Recent research has demonstrated that circulating OMVs containing gingipains may breach the blood-brain barrier (BBB) by invading microvascular endothelial cells and degrading tight junction proteins, leading to increased BBB permeability^24^. *P. gingivalis* has also been shown to increase BBB permeability via the major facilitator superfamily domain containing 2a /caveolin-1 (Mfsd2a/Cav-1) transcytosis pathway, potentially allowing *P. gingivalis* and/or gingipain virulence factors entrance to the brain^25^. Gingipains have previously been identified in the middle temporal gyrus (MTG) of Alzheimer’s disease (AD) brains, where gingipain load was shown to correlate with AD diagnosis and tau and ubiquitin pathology^26^. To date, the presence of gingipains in the substantia nigra pars compacta of PD patients has not been examined.

The current study reports on the identification, characterization, and ultrastructural localization of gingipains from *P. gingivalis* in the SNpc of PD patients and neurologically normal subjects using immunohistochemistry, immunofluorescence multi-labeling with confocal microscopy, and immunogold electron microscopy. Gingipains were identified within dopaminergic neurons of the SNpc in both neurologically normal subjects and PD patients, with gingipains occasionally colocalizing with phosphorylated- αSyn (αSynp) and Lewy bodies in the SNpc of PD patients. Proteomic analysis revealed that in vitro, αSyn is a target of Kgp proteolysis, generating aggregation-prone αSyn fragments. The results reported here do not demonstrate induction of PD by gingipains but rather should generate novel hypotheses for further mechanistic and functional studies on the role of *P. gingivalis* and gingipains in PD pathogenesis.

## Results

### RgpB and Kgp staining is abundant in neuromelanin-positive cells in the SNpc of control and PD brains

**Fig. 1:**
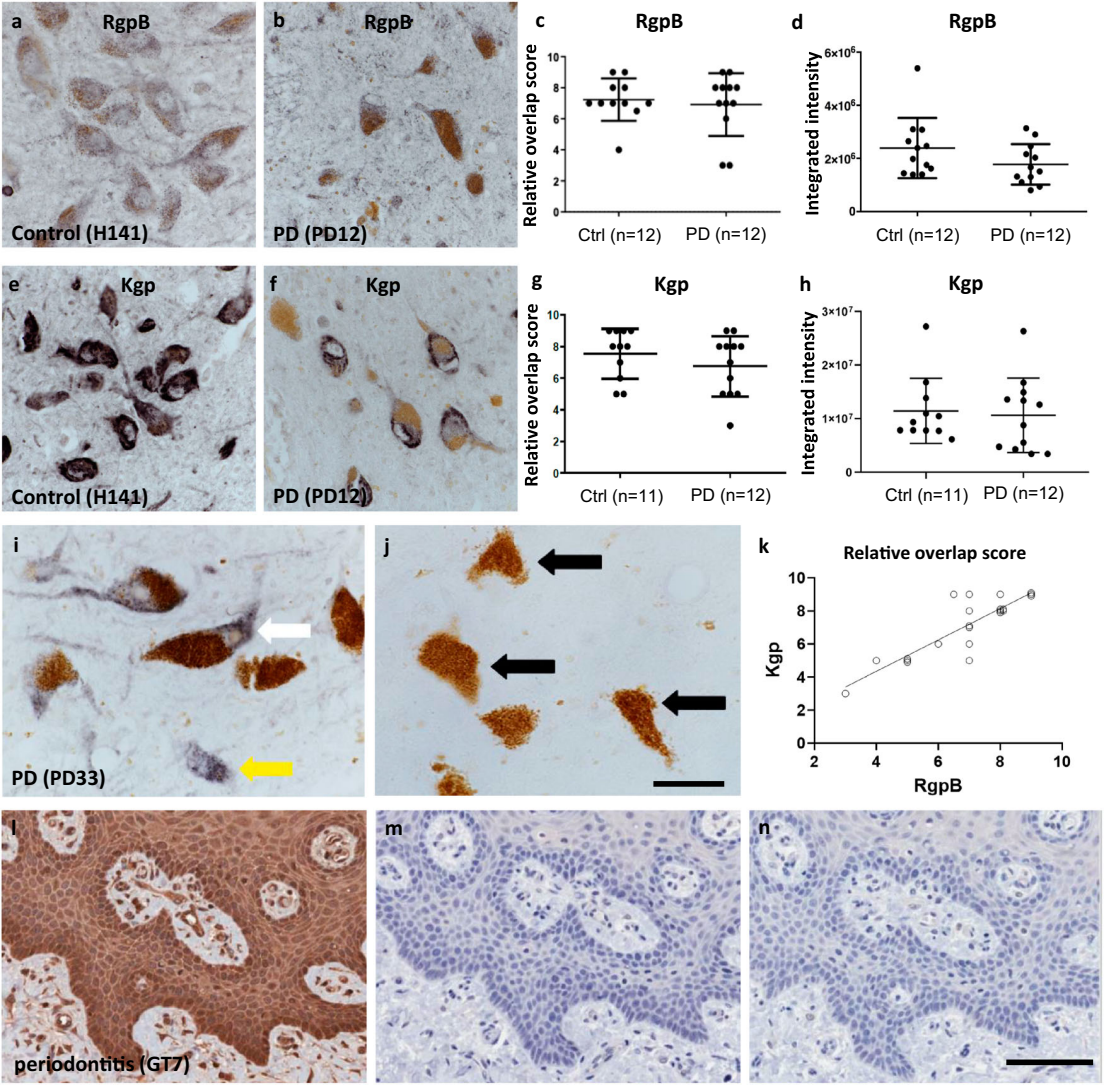
IHC staining of neuromelanin-containing neurons in the SNpc of control and PD brains. **a**, **b** CAB101 staining for RgpB in the SNpc revealed a granular cytoplasmatic stain (purple/black). Neuromelanin (brown) is clearly distinguishable from the purple black DAB-Ni reaction product in both control and PD patients. **c** Semiquantitative analysis of RgpB staining shows equal relative numbers of neuromelanin cells positive for RgpB in controls and PD patients. **d** Integrated intensity analysis of RgpB shows no difference between controls and PD (*t* test with Welch’s correction: *p* = 0.14). **e**, **f** CAB102.1 staining for Kgp in the SNpc revealed an intense cytoplasmatic stain (purple/black). Note the reduced density of neuromelanin cells in PD patients (**b**, **f**) compared to controls (**a**, **e**). **g** Semiquantitative analysis of Kgp staining shows equal relative numbers of neuromelanin cells positive for Kgp in controls and PD patients. **h** Integrated intensity analysis of Kgp shows no difference between controls and PD (*t* test with Welch’s correction: *p* = 0.58). **i**, **j** Specificity of CAB102.1 was tested by preabsorbing CAB102.1 with 10x concentration of Kgp antigen. Using non-absorbed antibody (**i**) both neuromelanin positive (white arrow) and neuromelanin negative (yellow arrow) cells were detected. In sections incubated with preabsorbed antibody no DAB-Ni reaction product was observed (**j**) and neuromelanin was observed as before (black arrows). The section shown is from the SNpc of a PD case (PD33, Table 2). Scale bar 20 µm. **k** The correlation of the relative overlap score of Kgp staining strongly correlated with RgpB staining (*r* = 0.864, *p* < 0.0001). **l**, **n** gingival tissue from a periodontitis patient (GT7) was used as a positive control for CAB102.1. **l** CAB102.1 at 1 µg/mL concentration. **m** CAB102.1 was substituted for recombinant rabbit IgG (1 µg/mL). **n** No primary antibody was used. Scale bar 100 µm. The scatter plots show the median with interquartile range (**c**, **g**) or the mean with standard deviation (**d**, **h**). The trendline in graph (**k**) represents a simple linear regression.

To assess the load of the *P. gingivalis*-derived gingipains RgpB and Kgp on neurons in the substantia nigra pars compacta (SNpc), polyclonal rabbit-derived antibody RgpB-specific CAB101 was used as previously described^26^, along with a newly manufactured Kgp-specific polyclonal antibody CAB102.1. CAB102.1 was produced using the same antigen and method as previously described for CAB102^26^. A titration series was performed and 1:1000 dilution was determined as the optimal concentration (data not shown). Using this concentration, specificity was confirmed by pre-absorbing the antibody solution with a 10x concentration of Kgp antigen. No staining was observed on slides incubated with pre-absorbed CAB102.1, indicating that the staining was specific for Kgp (Fig. 1i, j). As a positive control we stained a section obtained from gingival tissue of a periodontitis patient (Fig. 1l). The staining pattern was comparable to the Kgp in periodontitis controls staining observed in previous studies^26^. In addition, we performed recombinant IgG and no-primary controls (Fig. 1m, n). Immunohistochemical (IHC) staining was performed on paraffin sections of human SNpc of Parkinson’s disease (PD) patients (Table 2). Both CAB101 and CAB102.1 IHC processing resulted in granular and diffuse intracellular staining. Both RgpB and Kgp staining were clearly distinguishable from endogenous neuromelanin, which had a brown appearance compared to purple-black DAB-nickel reaction product used to visualize the antibodies (Fig. 1a, b, e, f). Immunohistochemical analysis was performed in the SNpc of 11 normal and 12 PD paraffin-embedded samples chosen based on an absence of neuropathology or a clearly defined PD neuropathology, respectively (Table 2). Three sections from each SNpc were cut, dewaxed and immunolabelled with either CAB101 or CAB102.1. RgpB staining was granular and occasionally diffuse, mostly localized to the cytoplasm with occasional punctate staining in the nucleus (Fig. 1a, b). Kgp staining was found at the same intracellular locations as RgpB, but with the staining much denser, resulting in a more solid staining pattern (Fig. 1e, f). Most of the staining was found in neuromelanin-positive neurons, but neuromelanin-negative/RgpB-positive or neuromelanin-positive/RgpB-negative cells were observed as well. The staining appeared equally intense in control or PD cases. However, as expected, the number of neuromelanin-positive cells was greatly reduced in PD cases. Quantification of neurons in the SNpc was beyond the scope of this study and a rough estimate by the investigators confirmed that in PD cases neuron density on the slides appeared to be reduced to less than 50% of the density observed on slides from control cases. To determine the load of RgpB and Kgp specifically in nigral dopamine neurons we performed a semiquantitative analysis, the “relative overlap score”, by scoring the overlap of RgpB and Kgp staining with neuromelanin-containing neurons. A scale of 0−10 was used, with zero indicating no overlap and 10 indicating complete overlap for all neuromelanin-containing dopamine neurons. No difference was observed in control versus PD cases in the relative number of neuromelanin-containing neurons positive for RgpB (Fig. 1c) or Kgp (Fig. 1g). These findings were confirmed when we performed an analysis of integrated intensity of RgpB and Kgp staining in neuromelanin neurons (Fig. 1d, h, respectively). Correlative analysis between the overlap score of Kgp and RgpB conducted on the pooled control and PD cohorts revealed a strong and highly significant correlation (Fig. 1k; *r* = 0.864, *p* < 0.00001). This correlation data further validates the gingipain IHC indicating a significant impact of *P. gingivalis* on the SNpc of control and PD included in this study.

### Alpha-synuclein is a substrate of Kgp and proteolysis with Kgp results in aggregation- inducing fragments

**Fig. 2:**
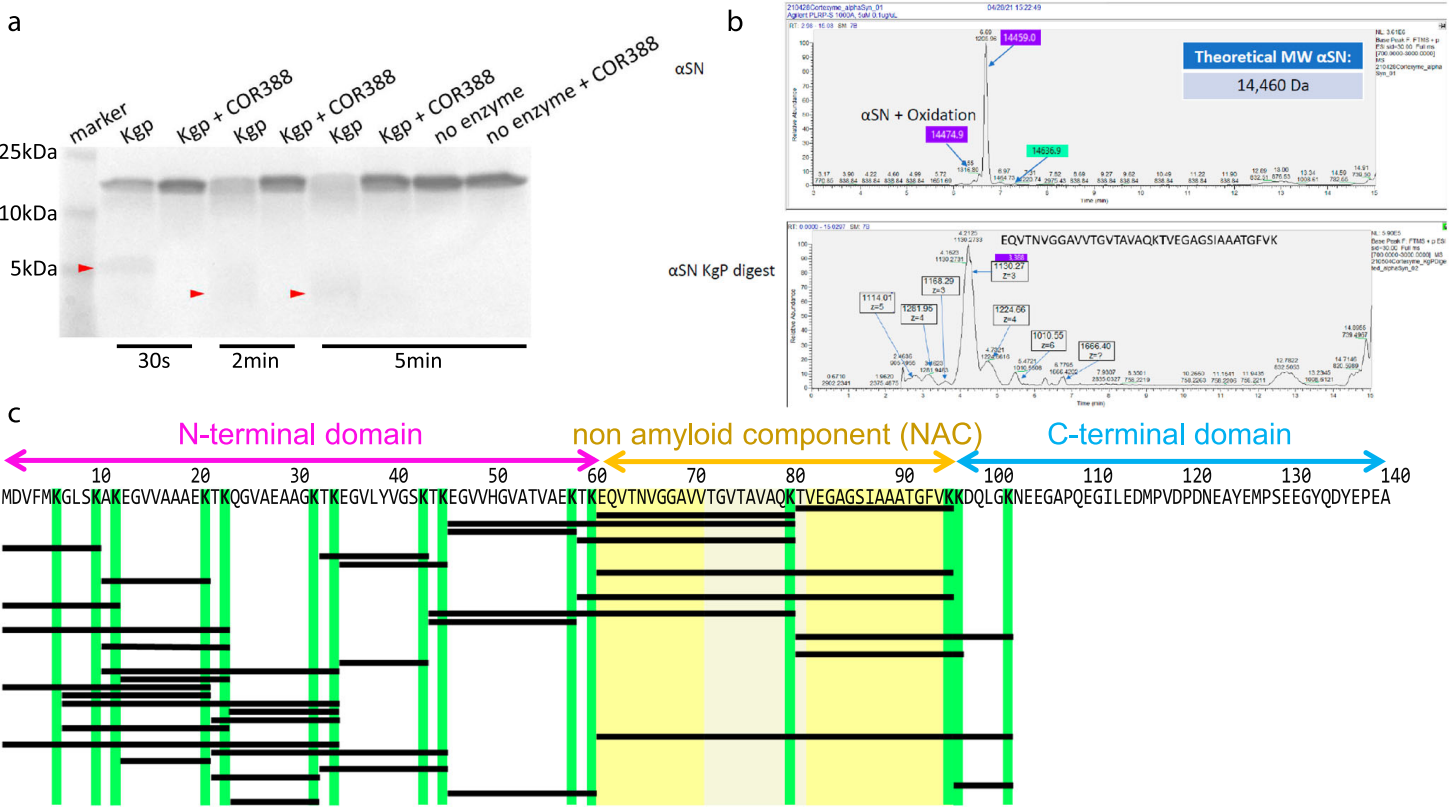
α-synuclein (αSyn) is a substrate for Kgp. **a** Coomassie gel of recombinant αSyn exposed to 30 s, 2 min or 5 min of Kgp, with or without Kgp inhibitor COR388. Fragmented αSyn appears as a faint diffuse smear at 5 kDa and below after 30 s of Kgp digestion and appears fainter with a wider spread at 2 min and 5 min digestion (red arrowheads). **b** Intact mass spectrometry analysis of recombinant non-digested and Kgp-digested αSyn. In the undigested sample a single peak was observed with a predicted mass matching the theoretical mass of αSyn. In the Kgp digested sample several peaks were observed with a dominant peak matching the sequence of the non-amyloid component (NAC)-domain of αSyn. **c** Amino acid sequence of αSyn. The three domains (N-terminal, NAC, C-terminal) are highlighted above the sequence. Kgp protease activity was observed after lysine (K; highlighted in green) resulting in fragments defined by the position of lysine (black bars). The NAC is flanked by lysine and Kgp activity resulted in several NAC specific fragments including the highly aggregating VTGVTAVAQKTV motif (grayed yellow).

Because of the abundance of gingipains observed in dopaminergic cells of the SNpc (Fig. 1a, b, e, f), and since αSyn truncation and fragmentation have been proposed to play roles in inducing formation of αSyn aggregates^27,28^, it was of interest to determine if αSyn is a target of gingipain proteolysis. Inspection of the amino acid sequence of αSyn revealed 15 lysine residues which are all potential points of protease activity of Kgp (Fig. 2c). Intriguingly, the non-amyloid component (NAC) domain, which includes amino acids (AA) 61−95, was observed to be immediately flanked by lysine residues at both the N-terminal and C-terminal (Fig. 2c). The NAC peptide fragment was initially identified in Alzheimer’s disease (AD) brains^29,30^ and was subsequently shown to promote aggregation of αSyn^31,32^. To evaluate if αSyn is a substrate for Kgp leading to generation of the NAC fragment, recombinant αSyn (rαSyn) was first exposed in vitro to Kgp and the integrity of the full-length rαSyn protein was analyzed on a Coomassie gel (Fig. 2a). When exposed to Kgp, a time-dependent digestion of rαSyn was found, while no degradation of the full-length protein was observed when Kgp was omitted or inhibited by adding COR388, a specific Kgp inhibitor^26^ (Fig. 2a). Of note, αSyn does not contain any arginine residues in its sequence and therefore would not be predicted to be a substrate of RgpB. In support of this, exposure of full-length rαSyn to RgpB had no effect on the integrity of the protein (supplementary Fig. 1). Next, protein fragments produced by Kgp digestion of rαSyn were analyzed by intact protein mass spectrometry (Fig. 2b, c, Table 1). As can be seen in the mass chromatogram in Fig. 2b, a peptide identified as the NAC domain was generated after full-length rαSyn was exposed to Kgp for 2 min. Several more peptides containing all or parts of the NAC domain were also identified (Table 1, underlined). Most of the peptides generated from the NAC domain contain the VTGVTAVAQKTV motif that has been found to be necessary and sufficient for αSyn fibrillization^31^ (Fig. 2c: grayed yellow; Table 1: bold letters).

**Table 1:** Peptides identified by intact mass analysis after proteolytic digestion of recombinant α-synuclein with Kgp

Peptide	Length	AA position	AUC
TVEGAGSIAAATGFVK	16	81−96	1.10E + 07
EQVTNVGGAVV**TGVTAVAQK**	20	61−80	8.30E + 06
EGVVHGVATVAEKTKEQVTNVGGAV**VTGVTAVAQK**	35	46−80	6.60E + 06
EGVVHGVATVAEK	13	46−58	6.30E + 06
TKEQVTNVGGAV**VTGVTAVAQK**	22	59−80	6.02E + 06
MDVFMKGLSK	10	1−10	4.41E + 06
TKEGVLYVGSK	11	33−43	3.89E + 06
EGVLYVGSKTK	11	35−45	3.59E + 06
EQVTNVGGAV**VTGVTAVAQKTV**EGAGSIAAATGFVK	36	61−96	2.94E + 06
AKEGVVAAAEK	11	11−21	2.69E + 06
TKEQVTNVGGAV**VTGVTAVAQKTV**EGAGSIAAATGFVK	38	59−96	2.67E + 06
MDVFMKGLSKAK	12	1−12	2.50E + 06
TKEGVVHGVATVAEKTKEQVTNVGGAV**VTGVTAVAQK**	37	44−80	2.50E + 06
TKEGVVHGVATVAEK	15	44−58	2.47E + 06
MDVFMKGLSKAKEGVVAAAEKTK	23	1−23	1.97E + 06
TVEGAGSIAAATGFVKKDQLGK	22	81−102	1.70E + 06
AKEGVVAAAEKTK	13	11−23	1.62E + 06
TVEGAGSIAAATGFVKK	17	81−97	1.59E + 06
EGVLYVGSK	9	35−43	1.51E + 06
AKEGVVAAAEKTKQGVAEAAGKTK	24	11−34	1.43E + 06
EGVVAAAEKTK	11	13−23	1.42E + 06
MDVFMKGLSKAKEGVVAAAEK	21	1−21	1.33E + 06
GLSKAKEGVVAAAEK	15	7−21	1.23E + 06
GLSKAKEGVVAAAEKTKQGVAEAAGKTK	28	7−34	1.20E + 06
QGVAEAAGKTK	11	24−34	1.15E + 06
TKQGVAEAAGKTK	13	22−34	1.14E + 06
GLSKAKEGVVAAAEKTK	17	7−23	1.01E + 06
EQVTNVGGAV**VTGVTAVAQKTV**EGAGSIAAATGFVKKDQLGK	42	61−103	9.15E + 05
MDVFMKGLSKAKEGVVAAAEKTKQGVAEAAGKTK	34	1−34	9.06E + 05
TKQGVAEAAGKTKEGVLYVGSKTK	24	22−44	7.54E + 05
EGVVAAAEK	9	13−21	7.17E + 05
TKEGVLYVGSKTK	13	33−44	6.86E + 05
TKQGVAEAAGK	11	22−32	5.19E + 05
KDQLGKN	7	97−103	4.44E + 05
EGVVHGVATVAEKTK	15	35−60	2.86E + 05
QGVAEAAGK	9	24−32	1.84E + 05
M( + 15.99)DVFMKGLSK^a^	10	1−10	1.59E + 05

The peptides are arranged in order of abundance indicated by AUC and correspond to fragments indicated by horizontal bars in Fig. 2c. Underlined sections indicate sequences that are part of the NAC and the aggregating VTGVTAVAQKTV motif is marked by bold font.

^a^oxidation at M1.

A total of 37 unique αSyn fragments were identified, confirming Kgp proteolytic activity on all 15 lysine residues in the full-length αSyn protein (Fig. 2c; Table 1). Of note, the NAC domain has a lysine in a central position which yields a 16 and a 21 AA fragment. These two fragments were found to be the most abundant in the intact protein mass spectrometry analysis (Fig. 2c; Table 1). Furthermore, the 16 AA peptide, αSyn 81−96, which was the most abundant peptide generated by Kgp, was recently identified in the cerebral spinal fluid of PD patients^33^.

### Immunofluorescence reveals RgpB and Kgp colocalize with phospho(S129)-alpha-synuclein and/or neuromelanin

**Fig. 3:**
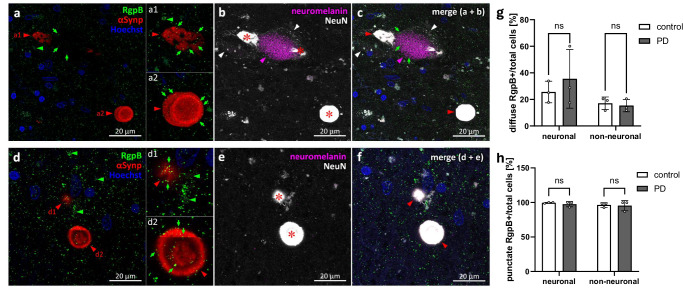
RgpB co-labeling using the CAB101 antibody (green) with αSynp (red), neuromelanin (magenta), and NeuN (white) in the PD SNpc. **a**−**c** RgpB shows punctate staining (green arrows) throughout the SNpc and within αSynp inclusions (red arrowheads) in neuromelanin-positive neuronal (**a1**) and neuromelanin and NeuN negative (**a2**) structures. **d**−**f** RgpB shows punctate staining (green arrows) within αSynp inclusions in neuromelanin negative/NeuN positive cells (**d1**, red arrowhead) and NeuN negative structures **(d2**, red arrowhead). Diffuse RgpB staining (green arrowheads) is observed outside of αSynp inclusions in neuromelanin negative/NeuN positive cells (**d1**, green arrowheads). αSynp staining bleeds through into the NeuN channel (**b**, **c**, **e**, **f**, red asterisk on saturated white) and was thresholded out for quantitative analysis of NeuN. Blue = Hoechst. **g**, **h** Quantification of the number of diffuse and punctate RgpB positive neuronal (NeuN positive) and non-neuronal cells relative to the total number of cells reveals no differences between neuronal or non-neuronal cells or between control or PD samples. Means with standard deviations, ns non-significant (*p* > 0.05, Tukey’s multiple comparison test). Scale bars are 20 µm.

The high levels of gingipains detected in the substantia nigra, the predominant area of PD-related αSyn aggregation, and the findings that exposure to Kgp results in aggregation-promoting fragments of αSyn, prompted an investigation to determine if there is an intracellular interaction between Kgp and αSyn. SNpc paraffin blocks from 3 control and 3 age-matching PD patients were selected for fluorescence multi-labeling and confocal microscopy analysis (Table 2, bold font). All sections were stained for either RgpB or Kgp and co-labeled with phospho(S129)-alpha-synuclein (αSynp), NeuN and Hoechst. Endogenous neuromelanin (NM) was detected by light absorption of transmitted light. αSynp has been established as the standard marker to differentiate pathogenic aggregated αSyn from non-pathogenic αSyn^34^. CAB101 labeling showed punctate and diffuse staining patterns of RgpB throughout the SNpc (Fig. 3a, d). The punctate pattern resulting from RgpB staining appeared uniformly distributed and did not appear to concentrate in cells or extracellular structures and nearly all cells were positive for punctate RgpB (99.7% controls and 97.1% PD; Fig. 3a, d, h). In contrast, diffuse RgpB staining pattern appeared to be intracellular with an average of 25% of neuronal (23.6 ± 7.2% controls vs 30.1 ± 18.2% PD) and 17% non-neuronal cells (17.7±% controls vs 15.9±% PD) were positive for diffuse RgpB staining (Fig. 3a, d, g). Neither punctate nor diffuse RgpB staining frequency was significantly different in PD cases vs controls (p-values of 0.63 and 0.67, respectively, Fig. 3g, h). αSynp aggregates for PD pathology were observed displaying both solid irregular aggregates (Fig. 3a1, d1) and more typical “donut” patterns with intense ring-like staining and a weaker stained core (Fig. 3a2, d2). These donut shaped structures were not associated with any cell-specific marker used in this study, and it is unclear if these are intracellular or extracellular aggregates. Punctate staining for RgpB frequently overlapped with αSynp staining and with endogenous neuromelanin (Fig. 3c) but there was no difference observed in the staining pattern of RgpB and αSynp between PD and controls. However, in SNpc tissues from controls, αSynp aggregates were rarely observed. Colocalization of punctate RgpB with αSynp was frequently observed, however there was no indication of an accumulation around αSynp (Fig. 3a2−d2). Diffuse RgpB was observed in cells with αSynp pathology, but there was no tendency for increased overlap with aSyn (Fig 3d1).

**Table 2:** List of neurologically normal and PD cases used

Case	Confirmed diagnosis	Age (years)	Sex	Post-mortem delay (hours)	Cause of death	Pathology - diagnosis	Use
H141	Neurologically normal	20	Male	22	Asphyxia	No significant histological abnormalities	FFPE; IHC
H144	Neurologically normal	76	Male	18.5	Ruptured aortic aneurysm	Non-specific age-related cerebral cortical change	FFPE; IHC,
H147	Neurologically normal	48	Male	17	Ischemic heart disease	No significant histological abnormalities	FFPE; IHC
H150	Neurologically normal	78	Male	11	Ruptured myocardial infarction	No significant histological abnormalities	FFPE; IHC
H180	Neurologically normal	73	Male	33	Ischemic heart disease	Control specimen: No significant pathological changes	FFPE; IHC
H190	Neurologically normal	72	Female	19	Ruptured myocardial infarction	Control specimen: Age-related microscopic changes	FFPE; IHC
H191	Neurologically normal	77	Male	20-25	Ischemic heart disease	No significant histological changes	FFPE; IHC
**H193**	**Neurologically normal**	**71**	**Male**	**23**	**Valvular heart disease/ coronary atherosclerosis**	**No significant histological changes**	**FFPE; IHC, IF, FF; TEM**
**H196**	**Neurologically normal**	**85**	**Male**	**15**	**Metastatic adenocarcinoma colon**	**No significant histological abnormalities**	**FFPE; IHC, IF, FF; TEM**
**H202**	**Neurologically normal**	**83**	**Male**	**14**	**Ruptured abdominal aortic aneurysm**	**No significant changes of degenerative type found. No LBD, No AD change (A0 B1 C0)**	**FFPE; IHC, IF, FF; TEM**
H204	Neurologically normal	66	Male	9	Ischemic heart disease	Appearance unremarkable for age	FFPE; IHC
PD10	Parkinson’s disease	70	Male	24	Pulmonary embolism	Idiopathic Parkinson’s disease	FFPE; IHC
PD11	Parkinson’s disease	69	Female	36	Perforated gastric ulcer and peritonitis	Idiopathic Parkinson’s disease	FFPE; IHC
PD12	Parkinson’s disease	76	Female	3	E.coli septicemia; myelodysplasia	Idiopathic Parkinson’s disease	FFPE; IHC
PD14	Parkinson’s disease	81	Male	11	Bronchopneumonia, obstructive jaundice, Parkinson’s disease, dementia	Idiopathic Parkinson’s disease	FFPE; IHC
PD23	Parkinson’s disease	78	Female	18.5	Pneumonia, 2o urinary tract infection, and Parkinson’s disease	Idiopathic Parkinson’s disease	FFPE; IHC
PD27	Parkinson’s disease	77	Male	4	End stage Parkinson’s disease/ cachexia	Parkinson’s disease	FFPE; IHC
PD28	Parkinson’s disease	76	Female	27	Bronchopneumonia/ congestive heart failure	Parkinson’s disease	FFPE; IHC
**PD31**	**Parkinson’s disease**	**67**	**Male**	**25**	**Respiratory failure**	**Parkinson’s disease**	**FFPE; IHC, IF, FF; TEM, 3D**
PD33	Parkinson’s disease/ cerebral Lewy body disease /Alzheimer’s disease	91	Male	4	Pneumonia	Idiopathic Parkinson’s disease, Lewy body disease, neocortical (diffuse), Alzheimer’s disease change (A3 B2 C2) intermediate Alzheimer’s disease change, focal cerebral amyloid angiopathy	FFPE; IHC
PD37	Parkinson’s disease/ cerebral Lewy body disease	81	Male	4	Parkinson’s disease	Idiopathic Parkinson’s disease, Lewy body disease, neocortical (diffuse)	FFPE; IHC
**PD42**	**Parkinson’s disease/ cerebral Lewy body disease**	**84**	**Male**	**21**	**Myocardial infarction**	**Idiopathic Parkinson’s disease, Lewy body disease, neocortical (diffuse)**	**FFPE; IHC, IF, FF; TEM, 3D**
**PD48**	**Parkinson’s disease/ cerebral Lewy body disease**	**84**	**Male**	**18-20**	**-**	**Idiopathic Parkinson’s disease, Lewy body disease, neocortical (diffuse), low Alzheimer’s disease change (A1 B1 C0)**	**FFPE; IHC, IF, FF; TEM, 3D**

Bold font: Samples selected for TEM, immunofluorescence confocal, and 3D analysis.

*FFPE* formalin fixed paraffin embedded (10 µm), *IHC* immunohistochemistry chromogenic quantification, *IF* immunofluorescence quantification, *FF* fixed-frozen (50 µm), *TEM* transmission electron microscopy, *3D* three-dimensional visualization.

**Fig. 4:**
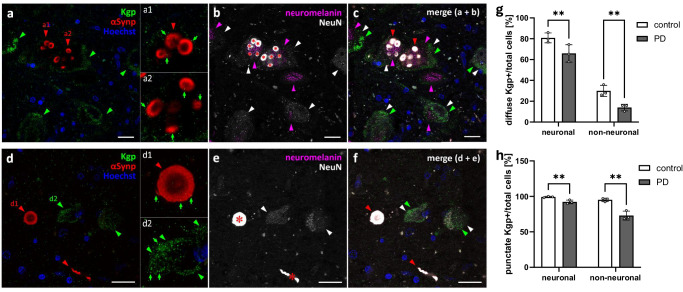
Kgp co-labeling using the CAB102.1 antibody (green) with αSynp (red), neuromelanin (magenta), and NeuN (white) in the PD SNpc. **a**−**c** Kgp shows diffuse staining (green arrowheads) in the cytoplasm of neuromelanin-positive (magenta arrowheads) and neuromelanin-negative neuronal cells (white arrowheads). **a1**−**a2** Punctate Kgp staining (green arrows) can be observed within αSynp inclusions (red arrowheads) in neuromelanin-positive neuronal cells. **d**−**f** Kgp shows diffuse staining (green arrowheads) within the cytoplasm of neuromelanin-negative neuronal cells (white arrowheads). Punctate Kgp staining (green arrows) can be observed within the periphery of αSynp structures (**d1**) as well as within the diffuse patches of Kgp staining in the cytoplasm of neuronal SNpc cells (**d2**). αSynp staining bleeds through into the NeuN channel (**b**, **c,** **e**, **f** red asterisk on saturated white) and was thresholded out for quantitative analysis of NeuN. Blue = Hoechst. **g**, **h** Quantification of the number of diffuse and punctate Kgp positive neuronal (neuromelanin and NeuN positive) and non-neuronal cells relative to the total number of cells reveals significantly more punctate and diffuse Kgp in control compared to PD samples. Means with standard deviations, **p < 0.01, Tukey’s multiple comparison test. Scale bars are 20 µm.

CAB102.1 labeling showed both punctate and diffuse staining patterns of Kgp within neuronal and non-neuronal cells (Fig. 4a−f). Punctate staining was observed in close proximity to, and occasionally overlapping with, the periphery of αSynp aggregates (Fig. 4a1, a2, d1). Comparable to RgpB, almost all neuronal cells (99.2 ± 1.0% in controls vs 92.2 ± 2.6% in PD) displayed a punctate staining pattern of Kgp (Fig. 4h), however the difference in PD vs controls proved to be significant (*p* < 0.01, Fig. 4h). Intriguingly, this difference in the relative number of cells positive for punctate Kgp was more pronounced in non-neuronal cells (95.2 ± 2.5% vs 73.0 ± 6.2%, in control vs PD, respectively). This pattern was mirrored in the analysis of diffuse Kgp positive cells. In neuronal cells it was found to be 80.8 ± 4.8% in control samples vs 65.9 ± 8.3% in PD samples. In non-neuronal cells 29.9 ± 5.3% vs 14.0 ± 3.2% were positive for diffuse Kgp (Fig. 4g). In addition to this remarkable decreased number of Kgp positive cells in PD samples, it is worth noting that the percentage of diffuse Kgp positive cells compared to diffuse RgpB is 3.2-fold higher in neuronal controls, 1.8-fold higher in non-neuronal controls, 1.9-fold higher in neuronal PD and nearly equal (0.9-fold) in non-neuronal PD samples. In contrast, in non-neuronal cells of PD samples, the percentage of punctate Kgp positive cells was lower than RgpB positive cells (by a ratio of 0.76) while equal percentages were observed for Kgp and RgpB in neuronal and non-neuronal control samples (ratio of 1.0 and 0.99 respectively) and of neuronal PD samples (a ratio of 0.95).

In summary, we observed widespread RgpB staining in the SNpc of both PD and control samples. RgpB didn’t show any specific affinity to αSyn aggregates, and no differences were found in the amount of RgpB in PD vs controls. In contrast, Kgp appeared to have an affinity to the peripheral edge of αSyn plaques, and most neurons were affected by diffuse Kgp staining with the percentage of cells affected by intracellular Kgp significantly decreased in PD compared to control samples. Thus, Kgp, rather than RgpB, might be promoting αSyn pathology and/or neuron loss in the SNpc of PD patients.

### Alpha-synuclein associated Kgp is mostly located on the periphery of aggregates, occasionally inside

**Fig. 5:**
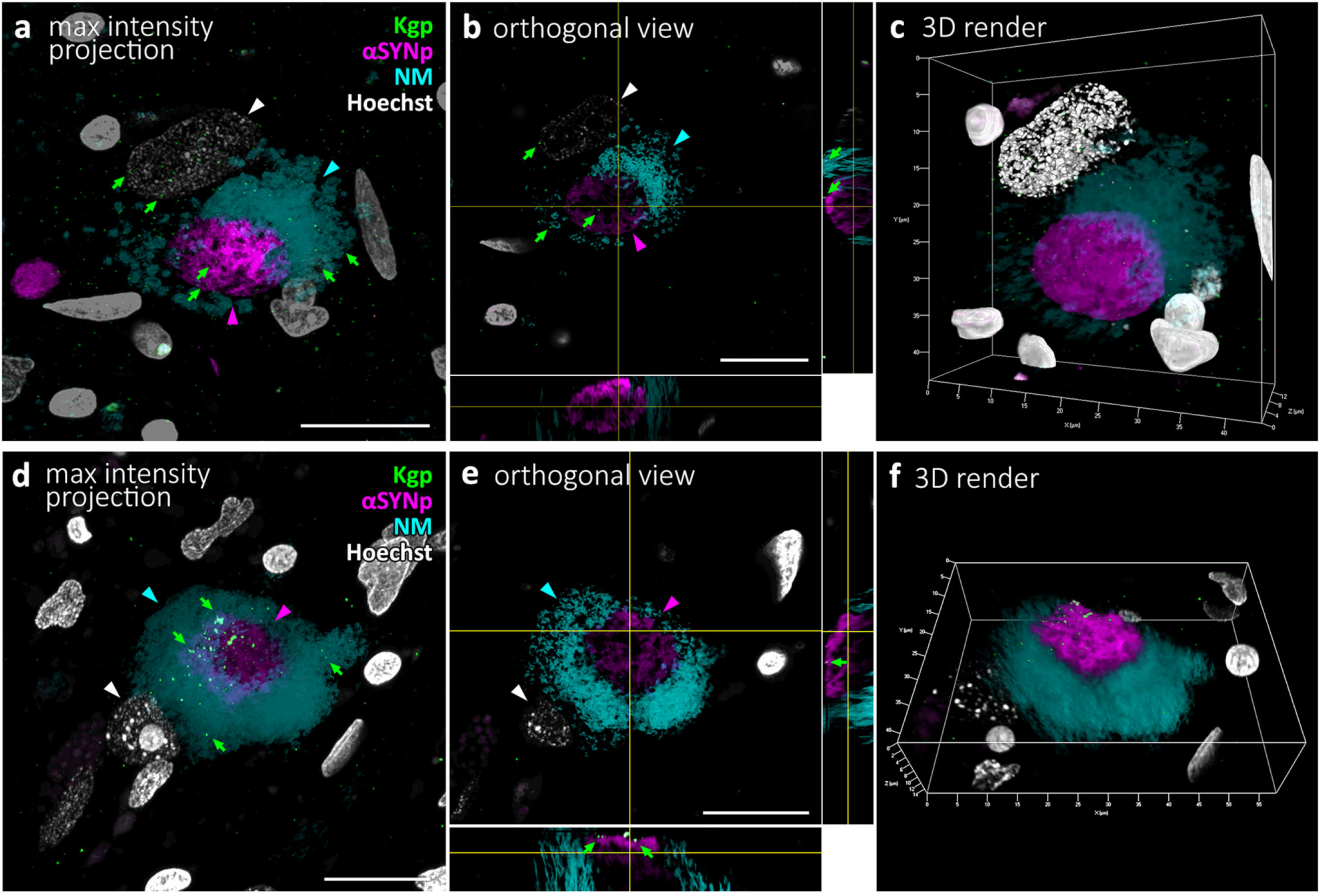
3D modeling of Kgp co-labeling using the CAB102.1 antibody (green) with αSynp (magenta), neuromelanin (cyan), and Hoechst (white) in two cells of the PD SNpc using confocal laser-scanning microscopy image stacks. **a** Maximal intensity projection of an image stack depicting a neuromelanin neuron. A few Kgp puncta (green arrows) can be seen within the neuromelanin and nucleus of the cell. **b** Orthogonal single plane view of the neuromelanin neuron from (**a**). The large nucleus (white arrowhead) can be seen in the white channel. A few Kgp puncta (green arrows) can be seen on the surface of the circular αSynp inclusion (magenta) as well as within the circular αSynp aggregate (magenta arrowhead). **c** 3D rendering of the same cell as in a-b shown in a 23-degree rotation around the Y-axis. **d** Maximal intensity projection of an image stack depicting a neuromelanin neuron. **e** Orthogonal 2-plane view of the neuromelanin neuron from d. A few Kgp puncta (green arrows) can be seen decorating the outside of the αSynp inclusion. **f** 3D rendering of the same cell as in (**d**, **e**) shown in a 32-degree rotation around the X-axis.

To better understand the intracellular relationship between Kgp, αSyn aggregates and neuromelanin, we collected confocal image stacks (Fig. 5a, d) of αSyn and neuromelanin positive neurons and analyzed 3D orthogonal (Fig. 5b, e) or surface rendered reconstructions (Fig. 5c, f) of the Kgp-stained sample set. Kgp positive granules were observed mostly on the surface of αSyn aggregates, but occasionally found embedded inside. In addition, Kgp was observed dispersed through neuromelanin and in the perinuclear cytoplasm.

### Ultrastructural analysis detects gingipains in mitochondria and nucleus and near aggregated alpha-synuclein

**Fig. 6:**
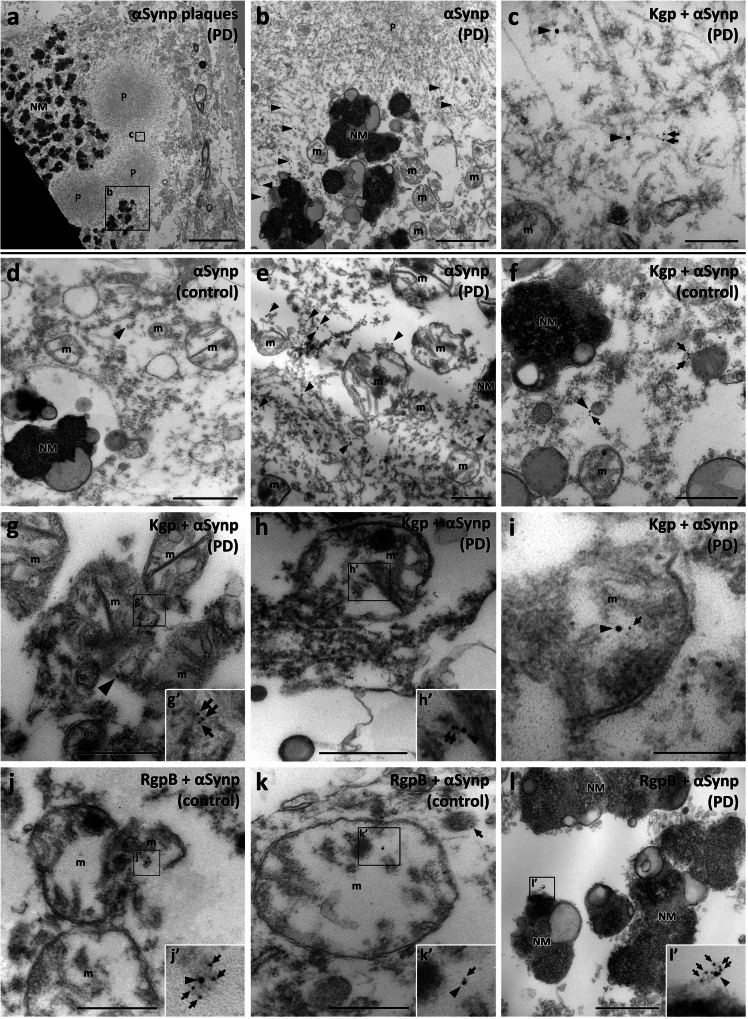
Transmission electron microscopy analysis in the SNpc using double immunogold labeling with CAB101 (anti-RgpB; 6 nm gold particles, arrows) or CAB102.1 (anti-Kgp; 6 nm gold particles, arrows), and anti-αSynp (15 nm gold particles, arrowheads). **a**−**c** Kgp and αSynp in association with αSynp plaques. **a** An overview image of a neuromelanin-positive cell with multiple circular αSynp plaques (P). **b** Enlarged region from (**a**). αSynp immunogold labeling (black arrowheads) can be seen on the periphery of the plaques and in proximity to neuromelanin (NM) granules. **c** Enlarged region from (**a**). αSynp (black arrowheads) and Kgp (black arrows) immunogold labeling can be seen on medium electron-dense tendrils at the periphery of the plaque. **d** A control case with a 15 nm gold particle (black arrowhead) marking the rare presence of αSynp in the cytoplasm of a neuromelanin cell. **e** More frequent labeling of αSynp was observed in PD cases (black arrowheads). **f** Kgp (black arrows) associated with αSynp (black arrowhead) in cytoplasm near neuromelanin (NM) and mitochondria (m) (**g**). **g** Kgp (black arrows) and αSynp (black arrowhead) in proximity and within a mitochondrion (m). **h**, **h’** Kgp (black arrows) associated with αSynp (black arrowhead) in a mitochondrion (**m**). **i** Kgp (black arrow) associated with αSynp (black arrowhead) in a mitochondrion (m). **j**, **j’** RgpB (black arrows) associated with αSynp (black arrowhead) in a mitochondrion (m). **k**, **k’** RgpB (black arrow) associated with αSynp (black arrowhead) in a mitochondrion (**m**). **l**, **l’** Rgp (black arrows) associated with αSynp (black arrowhead) on the surface of a neuromelanin granule. NM, neuromelanin; m, mitochondria, P, αSynp plaque. Scale bars: 5 µm (**a**), 1 µm (**b**, **d**), 500 nm (**e**, **f**, **g**, **h**, **j**, **k**, **l**, **m**), 200 nm (**c**, **i**).

**Fig. 7:**
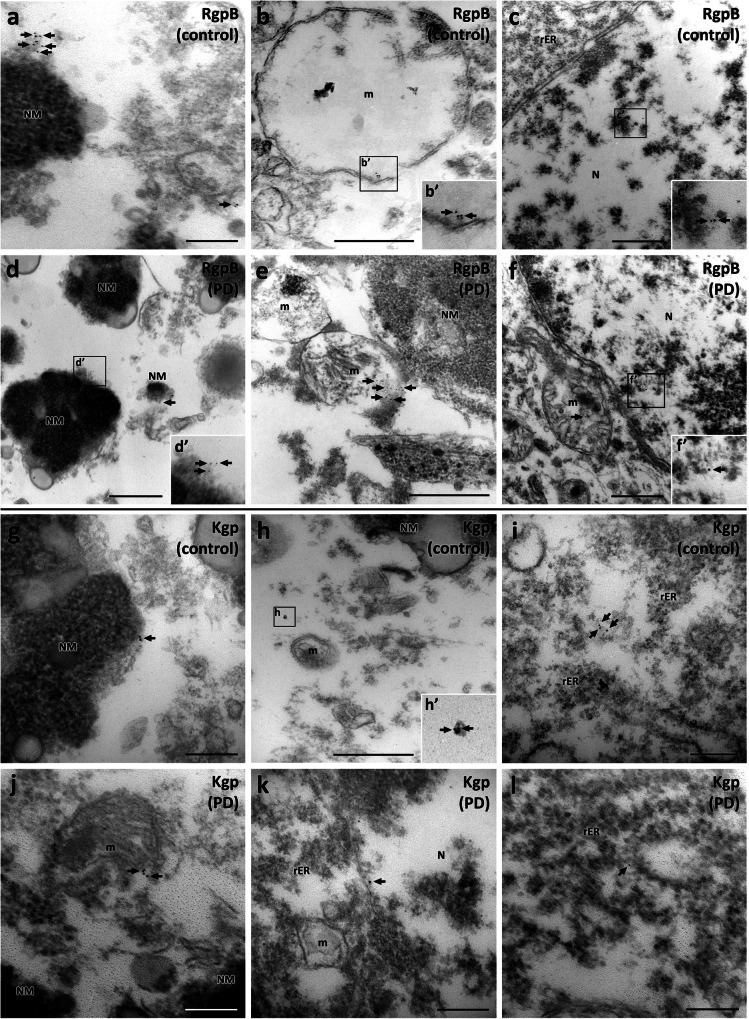
Immunogold labeling of RgpB and Kgp (6 nm gold particles) in control and PD neuromelanin-positive cells of the SNpc. A similar pattern of RgpB immunogold (black arrows) was observed in samples from control (**a**−**c**) and PD cases (**d**−**f**). Clusters of 6 nm gold particles labeling RgpB were observed in association with neuromelanin (**a**, **d**), mitochondria (**b**, **e**), and the nucleus (**c**, **f**). The pattern of Kgp immunogold (black arrow) was comparable in control (**g**−**i**) and PD cases (**j**−**l**). Clusters of 6 nm gold particles labeling Kgp were observed in association with neuromelanin (**g**), mitochondria (**h**, **j**), the nucleus (**k**), and rough endoplasmic reticulum (**i**, **l**). NM: neuromelanin, m: mitochondria, P: αSynp plaque, PD: Parkinson’s disease, rER; rough endoplasmic reticulum. Scale bars: 500 nm (**b**, **c**, **d**, **e**, **f**, **h**), 200 nm (**a**, **g**, **i**, **j**, **k**, **l**).

To obtain an understanding of gingipain-αSyn interaction at the ultrastructural level, we performed dual immunogold co-labeling for αSynp and RgpB or Kgp. While postprocessing of formalin-fixed frozen tissue for transmission electron microscopy (TEM) and immunogold labeling is not ideal and can result in some structural loss and deformation, we found the tissue integrity to be of sufficient quality for immunogold staining and identification of the relevant ultrastructure such as nuclei, nuclear membranes, and mitochondria. Positive and no primary control experiments were performed to control for non-specific immunogold signals (Supplementary Fig. 3). A total of 136 neuromelanin positive cells from the 3 control and 3 PD cases included in the TEM study (Table 2) were examined. 35% of these cells had evidence for both αSynp and RgpB (22/63) and 36% had evidence for both αSynp and Kgp (26/73). As reported by others, αSyn plaques could be identified as round structures with an electron dense core and a more electron lucid perimeter that consisted of tightly packed fibrils (Fig. 6a). Phosphorylated αSyn was observed in the peripheral fibrils of these plaques and extended out into neuromelanin deposits and mitochondria (Fig. 6b). Immunogold labeling for Kgp was observed in the vicinity of αSynp fibrils (Fig. 6c) and was also located in or near mitochondria, and neuromelanin (Fig. 6d−i). Immunogold particles indicating the presence of αSynp were found in both control and PD cases (Fig. 6d, e respectively). αSynp was observed much less frequently in controls, but the intracellular locations were comparable in control and PD cases (Fig. 6d, e). αSynp was observed in the vicinity of mitochondria, occasionally appearing to be inside mitochondria, near neuromelanin, and near fibrils of medium electron density (Fig. 6d, e). Immunogold particles against gingipains were frequently observed in these same locations and were often closely associated with αSynp (Fig. 6f−l). This same association of gingipains with neuromelanin and mitochondria was also frequently observed without the presence of αSynp (Fig. 7). Additionally, RgpB or Kgp was also observed in association with other cell compartments such as the nucleus (Fig. 7c, f) and the rough endoplasmic reticulum (Fig. 7k, l). No difference was apparent in the pattern or intracellular localization of RgpB or Kgp in control compared to PD cases.

## Discussion

This study offers evidence that gingipains from *P. gingivalis* are found in neuromelanin positive neurons in the SNpc of the human brain. In the SNpc of both control and PD brains, approximately 75% of all dopaminergic neurons were gingipain positive. This data demonstrates that gingipains have a strong affinity to accumulate inside neuromelanin-positive neurons of the SNpc. Interestingly, the immunofluorescence analysis confirmed the nearly ubiquitous presence of gingipains in the brain area investigated. However, there appeared to be a differential association of RgpB and Kgp with αSyn, with no association of RgpB with αSyn aggregates, and an equal percentage of neuronal and non-neuronal cells positive for RgpB. In contrast, Kgp was found in the periphery of αSyn aggregates, a finding confirmed in the 3D reconstruction analysis and in the TEM analysis. Furthermore, the percentage of cells that colocalized with Kgp was significantly decreased in PD samples. This might seem counterintuitive, but Kgp has been shown to be directly neurotoxic^26^, and the number of neurons in the SNpc of PD patients is reduced to less than 50% of controls. Therefore, a hypothetical explanation for the decreased number of Kgp-positive neurons might be that in PD these neurons are more sensitive to Kgp and have died off, leaving a greater relative percentage of Kgp-negative cells in PD patients.

It is noteworthy that a majority of neurons appeared capable of surviving chronic exposure to gingipains as observed in control brains, and to a similar degree in the epithelial cells of gingival tissue (Fig. 1l). However, it is known that *P. gingivalis* has mechanisms to prevent cells it has infected from undergoing apoptosis^35^. We thus hypothesize that a number of factors might be necessary to act in concert to result in the death of dopaminergic neurons by gingipains, since the difference between control and PD patients does not appear to manifest in increased accumulation of gingipains but might rather be the result of a heightened vulnerability to the presence of gingipains through one of the mechanisms discussed below.

In addition to the loss of Kgp-positive neurons, other possibilities exist for the decrease in immunogenicity of Kgp in PD brains, including Kgp export from the SNpc, degradation, or modification of the antibody epitope as the disease progresses into the symptomatic stage and αSyn pathology accumulates. Since Kgp, but not RgpB, fragments αSyn, and proteolytic processing of αSyn has been linked to αSyn aggregation^28^, one hypothetical scenario is that Kgp is trapped within αSyn aggregates as they form, perhaps masking the Kgp antibody epitope in such a way that the antigen is not retrievable during the IHC process. Further studies examining the relationship between Kgp cleavage of αSyn and formation of αSyn aggregates that may trap Kgp are warranted.

In a previous Alzheimer’s disease (AD) study^26^ we also observed extensive gingipain labeling in control brains, 39% were positive for RgpB and 52% for Kgp, though the area investigated was the MTG which contains marginal numbers of neuromelanin-positive neurons. The main finding in the AD study was a significantly increased gingipain load in the MTG of AD patients compared to controls that correlated with tau and ubiquitin pathology and a small cohort of PD patient samples was included that had an RgpB load in the MTG comparable to the control group. However, the methodology and brain area analyzed are different than in the current study and we would caution to draw many conclusions comparing the two data sets. The risk of developing PD is strongly age dependent and our identification of gingipain antigens in the SNpc of individuals without PD with little neuron loss or accumulation of αSyn aggregates argues that the presence of gingipains is an early event that may take years if not decades to manifest in PD pathology in susceptible individuals if they live long enough. *P. gingivalis* infection is highly prevalent in the human population, with one Finnish study showing that salivary carriage of *P. gingivalis* is significantly associated with age, increasing from 13% salivary carriage in subjects 30- to 34-years-old, to 56% in subjects 60- to 64-years-old^36^.

In the SNpc of PD subjects, 3D reconstructions of αSyn aggregates with Lewy body morphology confirmed the presence of Kgp predominantly along the periphery of aggregates, suggesting that Kgp might be involved in Lewy body formation. In this regard, in vitro proteomic analysis revealed that αSyn is a target of Kgp proteolysis, generating multiple αSyn fragments, including the NAC fragment that has been shown to be associated with Lewy body structure (Moors 2021)^37^. Unlike Kgp, RgpB was unable to cleave αSyn in vitro due to the lack of target arginine residues in the αSyn protein.

Importantly, immunogold electron microscopy revealed gingipain antigens within mitochondria of dopaminergic neurons in the SNpc in both control and PD subjects. Mitochondrial dysfunction has been reported to occur early in PD pathogenesis, prior to neuronal loss and prior to the emergence of αSyn pathology^38^. Gram-negative bacteria are known to target host mitochondria by releasing outer membrane vesicle (OMV) carrying mitochondrial toxins^39^. The small clusters of both RgpB and Kgp antigens identified near and within mitochondria by immunogold electron microscopy may represent remnants of *P. gingivalis* OMVs carrying gingipains. *P. gingivalis* OMVs were recently shown to cross the blood brain barrier and accumulate in the brain after oral administration in wild-type mice^40^.

Immunogold electron microscopy demonstrated the colocalization of RgpB and Kgp with αSyn inside of mitochondria. αSyn has previously been shown to be imported into mitochondria, with the first 32 amino acids of αSyn functioning as a cryptic mitochondrial targeting sequence^41^. Research has demonstrated a basal level of αSyn in mitochondria in the SNpc, striatum, and cerebellum of non-PD subjects^41^. How gingipains enter mitochondria requires further research, but a gingipain adhesion domain known as A44 was previously shown to be imported into mitochondria^35^. Also, further research will be required to determine if αSyn within mitochondria is cleaved by intra-mitochondrial Kgp, and what the consequences might be for maintaining basal levels of mitochondrial αSyn under Kgp-induced proteolytic stress, e.g., does the cell have to produce more αSyn and increase importation into mitochondria to maintain homeostasis. In this regard, intra-mitochondrial levels of αSyn in the SNpc and striatum of PD subjects have been reported to be abnormally elevated, with a 2- to 15-fold increase compared to non-PD subjects^41^.

*P. gingivalis* infection has previously been shown to cause mitochondrial dysfunction by increasing dynamin-related protein 1 (Drp1)-dependent mitochondrial fission (fragmentation) in endothelial cells^42^. Drp1 primarily resides in the cytosol and is translocated to mitochondria under stress conditions where it induces mitochondrial fragmentation leading to mitochondrial depolarization^43^. In a 1-methyl-4-phenyl-1,2,3,6-tetrahydropyridine (MPTP) animal model of PD, MPTP-induced Drp1 translocation to mitochondria increased mitochondrial fission and degeneration of dopaminergic neurons^44^. Research is needed to determine if gingipains or other *P. gingivalis* virulence factors may increase Drp1 activity in dopaminergic neurons.

In addition, *P. gingivalis* infection has been shown to impair the expression of the canonical PD-related proteins PTEN-induced putative protein kinase 1 (PINK1) and Parkin, two proteins critically involved in clearing damaged or dysfunctional mitochondria by mitophagy^45^. Mutations in *PINK1* and *PARK2* (the gene for Parkin) are involved in PD pathogenesis^46^. In *P. gingivalis-* infected bone marrow-derived macrophages, infection resulted in decreased mRNA transcription and protein expression of both PINK1 and Parkin, leading to an inhibition of mitophagy and a resultant accumulation of damaged mitochondria and reactive oxygen species (ROS)^45^. Further research is needed to determine the mechanism by which *P. gingivalis* manipulates PINK1 and Parkin expression, and if this inhibitory effect on mitophagy can occur in dopaminergic neurons.

Another potential target of gingipains in the SNpc is transferrin, a glycoprotein involved in iron transport that possesses two iron-binding sites, a property that allows transferrin to function as an antioxidant since the bound iron cannot act as a catalyst for the formation of toxic hydroxyl radicals^47,48^. Both RgpB and Kgp have been shown to cleave transferrin into fragments of various sizes, generating free iron and iron-containing transferrin fragments capable of catalyzing the formation of hydroxyl radicals^49^. In 2009 it was reported that dopaminergic neurons in the substantia nigra contain a novel mitochondrial transferrin/transferrin receptor 2-mediated iron transport pathway that delivers iron to mitochondria, and that this iron transport pathway is disrupted in PD^50^. In support of the 2009 study, a recent study found that transferrin was decreased in PD SN^51^. Whether or not gingipains identified in the SNpc in the current study cleave transferrin and disrupt mitochondrial iron homeostasis is a focus of future studies.

This study has limitations, with the major limitation being the small sample size. Since all the neurologically normal controls and PD subjects had evidence of both Kgp and RgpB antigens in the SNpc, a much larger sample size from different age groups and geographic regions is needed to determine if the accumulation of gingipains in the SNpc is a common phenomenon related to age.

Another limitation of the current study is the lack of genetic information on the subjects. It would be informative to know if any of the subjects carried genetic mutations or polymorphisms in *PINK1* or *PARK2,* since these genes would be critical in maintaining mitochondrial integrity in the presence of gingipain-induced proteolytic stress.

In the current study we were only able to analyze the SNpc. Matching tissue samples from the same patients would allow us to investigate if there is a preference for gingipains to accumulate in specific brain regions and if there are any correlations with peripheral tissues. In addition, future studies would benefit from the analysis of gingipains in peripheral tissues known to harbor αSyn pathology, including submandibular gland^52^, esophagus^53^, stomach^54^, vermiform appendix^55^, colon^56^, and peripheral nerve^57^.

The specificity of antibodies against RgpB (CAB101) and Kgp (CAB102.1) is essential for the findings presented. We have tested specificity by including positive biological control sections as well as controls for nonspecific secondary signal by omission of the primary antibodies for our immunohistochemistry and immunogold experiments. In addition, the antibody signal for RgpB and Kgp shows a strong correlation of overlap (Fig. 1k). RgpB and Kgp are often colocalized on the surface of *P. gingivalis* and its OMVs and this is a good indicator that the staining is specific.

In summary, the findings of this study offer evidence that gingipains from *P. gingivalis* may accumulate in the SNpc of the human brain. The reasons for this are currently unclear, as well as the mechanism for gingipain translocation from the periphery to the SNpc. One of the most intriguing findings of the current study was the observation of gingipains within mitochondria of dopaminergic neurons, and the detection of gingipain colocalized with intra-mitochondrial αSyn. However, discerning correlation from causation is now the major task in determining if gingipains play a role in triggering loss of dopaminergic neurons. As others have pointed out, most PD patients will likely have been exposed over decades to multiple pathogens associated with neurodegeneration, and therefore identifying a specific infectious agent involved in PD pathogenesis is extremely difficult^58^. Accordingly, studying the role of *P. gingivalis* and gingipains in the degeneration of SNpc dopaminergic neurons in PD animal models, such as the recently published oral *P. gingivalis* infection of *LRRK2* R1441G mice^7^, will be important, along with controlled cellular studies such as *P. gingivalis* infection or *P. gingivalis* OMV invasion of neurons derived from human induced pluripotent stem cells^59^. Ultimately, a pharmacologic approach using high-precision treatments against specific pathogens or their virulence factors in human PD clinical trials may be the best approach to determine causation. In this regard, high-precision Kgp inhibitors have entered human clinical trials for the treatment of mild to moderate dementia due to *P. gingivalis* infection^60^, and this same approach could be used in PD clinical trials where there is biomarker evidence of *P. gingivalis* infection.

## Methods

### Samples

Post-mortem human brain tissue was obtained from the Neurological Foundation Human Brain Bank at the University of Auckland, Centre for Brain Research (Table 2). The human tissue was donated to the Brain Bank with consent from the donors’ families and its use in this project was approved by the Health and Disability Ethics Committee. The normal cases had no clinical history of neurological disease and no significant pathological abnormalities upon post-mortem examination. The PD cases had a disease duration ranging from 9 to 23 years with an average of 16 years. Although the post-mortem delay of the normal cases was on average higher than the PD cases, it did not impact detection of phosphorylated α-synuclein or any of the other markers used in this study. Pathological examination by a neuropathologist confirmed the clinical diagnosis of PD by observed presence of Lewy bodies in the substantia nigra as well as pigment and cell loss in the substantia nigra. Gingival tissue samples were collected from human volunteers with chronic periodontal disease who provided signed informed consent after the nature and possible consequences of the studies were explained under a University of California at San Francisco IRB-approved protocol (approval no. 11-05608).

Neurologically normal controls (*n* = 11) and PD (*n* = 12) human substantia nigra tissue were used in these studies (Table 2). Cases were age and sex matched with an average age of 79.7 + /− 7.2 and 78.3 + /− 9.8 (*P* < 0.89) years in normal and PD respectively. Paraffin embedded tissue was sectioned at a thickness of 10 μm on a rotary microtome (Leica Biosystems, RM2235), floated in a water bath (38−42 °C; Leica Biosystems, HI1210), collected on UberFrost slides and air-dried at room temperature for 48 h. Fixed-frozen tissue was sectioned at 50 μm thickness on a Microm HM450 microtome and stored in 24-well plates immersed in PBS containing 0.1% azide. For in-depth analysis by TEM and multi-fluorescence 3D confocal microscopy, 3 PD and 3 control cases were selected based on availability of both paraffin and fixed-frozen tissue, and additionally age matched (Table 2, bold font).

### Antibody production

Polyclonal antibody CAB102.1 was produced by GenScript USA Inc. (New Jersey) using their express immunization protocol. The immunogen, amino acids 22−400 of Kgp (GenBank: BAG34247.1), was expressed in a bacterial system and used to immunize four rabbits over four consecutive immunizations. After the final immunization, the sera were pooled, and antigen affinity purified. Specific binding was validated by Western blots. Specific and nonspecific binding to human histology sections was controlled by pre-incubating the polyclonal antibodies with their respective immunogens before immunohistochemistry staining and positive control staining a gingival tissue form a periodontitis patient (Fig. 1g−j).

### Formalin-fixed paraffin embedded (FFPE) sections for chromogenic labeling

Sections were placed on the hot plate for 1 h at 60 °C before two xylene immersions (1 h and 10 min, respectively) for deparaffinization. The sections were subsequently rehydrated through a series of ethanol immersions (100% 2 × 5 min, 95% 2 min, 85% 2 min, 75% 2 min), and three 5 min milliQH_2_O water washes. Heat-induced antigen retrieval (HIER) was conducted in a pressure cooker (Model 2100-retriever; Pick Cell Laboratories) at 121 °C for 20 min, followed by a 2-h cool-down period. Following HIER, three milliQH_2_O water washes (5 min each) were conducted before a 20-min endogenous peroxidase block (50% MeOH, 1% H_2_O_2_, diluted in milliQH_2_O). Subsequently, the sections were washed in phosphate-buffered saline (PBS; 3 × 5 min). The following blocking steps and incubations were conducted in a humidity chamber to prevent the tissue from drying out. The sections were exposed to a blocking buffer (10% normal goat or donkey serum diluted in PBS) for 1 h at room temperature. After blocking, the sections were incubated with primary antibody solution at 4 °C overnight. The following day, the sections were washed in PBS with 0.2% Triton X-100 (PBS-T; 5 min), followed by 2 × 5 min PBS washes, before incubation in secondary antibody at room temperature for 3 h. The sections were washed in PBS-T and PBS, before ExtrAvidin®-Peroxidase incubation at room temperature for 1 h. Washing steps were conducted again prior to section incubation in the peroxidase substrate (0.5% 3,3′-diaminobenzidine (DAB), 0.01% H_2_O_2_ intensified with 0.04% nickel ammonium sulfate). The peroxidase substrate was washed off in 3 PBS and 3 milliQH_2_O washes. The sections were subsequently dehydrated in a graded ethanol series (75% 2 min, 80% 2 min, 95% 2 min, 100% 2 × 5 min), and cleared in xylene (3 × 10 min) before a coverslip was applied using a DPX mounting medium (DPX).

### Formalin-fixed paraffin embedded (FFPE) for immunofluorescence labeling

Sections were dewaxed for 1 h at 60 °C followed by immersion in room temperature xylene (2 × 20 min) and rehydrated through a series of ethanol (100% 2 × 10 min, 95% 5 min, 85% 5 min, 75% 5 min), and water (5 min). After heating in a pressure cooker (2100 Retriever) to 121 °C, the slides were left to cool for 2 h in an antigen retrieval buffer (10 mM sodium citrate buffer, pH 6). Once cooled, the sections underwent formic acid (80%) antigen retrieval for 4 min and were then washed in ddH_2_O followed by 3 × 10 min wash with PBS with 0.2% Tween20 (PBST). The following blocking steps and incubations were conducted in a humidity chamber to prevent the tissue from drying out. The slides were then incubated for 1 h with 10% normal goat serum (NGS) in 0.2% Triton X100 before being incubated with primary antibody solution of NeuN, anti-αSynp, and either CAB101 (ant-RgpB), or CAB102.1 (anti-Kgp) (Table 3) diluted in 1% normal goat serum in 0.2% Triton X100 for 24 h at 4 °C. A separate series of sections were incubated with a 1% NGS with 0.2% Triton X100 with the primary antibodies omitted as a negative control. The slides were washed in PBS then incubated with AlexaFluor conjugated secondaries and Hoechst (Table 3) diluted in 1% NGS with 0.2% Triton X100 for 2 h at room temperature. The sections were then washed in PBS and cover slipped with ProLong Gold antifade mounting medium and stored at 4 °C for imaging.

**Table 3:** IF: immunofluorescence quantification, IHC: immunohistochemistry, 3D: three-dimensional visualization, TEM: transmission electron microscopy

Antibody/chemicals/detection	Source	Identifier	Dilution
CAB101 RgpB, Rabbit (0.5 mg/ml)	Cortexyme, Inc.		IHC; 1:1000 IF, 3D, TEM; 1:500
CAB102.1 Kgp, Rabbit (0.46 mg/ml)	Cortexyme, Inc.		IHC; 1:1000 IF, 3D, TEM; 1:500
Anti-Alpha-synuclein (phosphoS129) antibody [P-syn/81 A], Mouse monoclonal	Abcam	Cat# ab184674, RRID:AB_2819037	IF, 3D, TEM; 1:3,000
Anti-NeuN purified, Guinea Pig polyclonal	Millipore	Cat# ABN90P, RRID:AB_2341095	IF; 1:250
biotinylated Goat anti-Rabbit	Thermo Fisher Scientific	Cat# A-16100	IHC; 1:1000
biotinylated Donkey anti-Rabbit	Thermo Fisher Scientific	Cat# A-16027	IHC; 1:1000
Goat anti-Rabbit IgG (H + L) Highly Cross-Adsorbed Secondary Antibody, Alexa Fluor 488	Thermo Fisher Scientific	Cat# A-11034, RRID:AB_2576217	IF, 3D; 1:250
Goat anti-Mouse IgG (H + L) Highly Cross-Adsorbed Secondary Antibody, Alexa Fluor 594	Thermo Fisher Scientific	Cat# A-11032, RRID:AB_2534091	IF, 3D; 1:250
Goat Anti-Guinea Pig IgG (H + L) Highly Cross-adsorbed Antibody, Alexa Fluor 647 Conjugated	Molecular Probes	Cat# A-21450, RRID:AB_141882	IF, 3D; 1:250
Hoechst 33342, Trihydrochloride, Trihydrate	Invitrogen Molecular Probes	Cat# H1399	IF, 3D; 1:20,000
ExtrAvidin−Peroxidase buffered aqueous solution	Sigma-Aldrich	Cat#E2886-1ML	IHC; 1:1000
Goat anti-Rabbit IgG (H + L) coupled with 6 nm gold, EM grade	Electron Microscopy Science, Auron 106.011	Cat# 25103	TEM; 1:50
Goat anti-Mouse IgG (H + L) coupled with 15 nm gold, EM grade	Electron Microscopy Science, Auron 115.022	Cat# 25132	TEM; 1:50

### Formalin-fixed frozen (FF) sections for 3D confocal microscopy analysis

Free-floating PD SNpc sections (bold font in Table 2) were permeabilized with PBS with 0.2% Tween20 (PBST), blocked in 5% NGS for 1 h, and then incubated with CAB102.1 and anti-αSynp antibodies diluted in 1% NGS with 0.2% Triton X100 (Table 3) for 72 h at 4 °C. Sections were then incubated with AlexaFluor conjugated secondaries and Hoechst diluted in 1% NGS with 0.2% Triton X100 overnight (Table 3). Washes between incubations was carried out with PBS. Sections were then mounted onto glass slides and cover slipped with Prolong Gold antifade mounting medium.

### Formalin- fixed frozen (FF) sections for transmission electron microscope (TEM) analysis

Free-floating control and PD SNpc sections (bold font in Table 2) were blocked in 5% NGS for 1 h and then incubated with CAB101 or CAB102.1 and anti- αSynp (Table 3) diluted in 1% NGS for 72 h at 4 °C. Immunogold detection of the target antigen was carried out with nano gold conjugated secondary antibodies (Table 3) diluted in 1% NGS overnight at 4 °C. Washes between incubations was carried out with PBS. Triton was omitted from all steps. From each section, 3 - 6 1 mm^2^ samples were dissected from nigral regions rich in pigmented neuromelanin nigral cells. These were transferred to glass scintillation vials and washed with 0.1 M phosphate buffer at 4 °C overnight before post-fixation in 1% osmium tetroxide for 1 h at 4 °C. The tissue was then dehydrated at room temperature with a graded alcohol series for five minutes each in two 70%, one 85%, one 95%, and four 100% alcohol changes. The fixed tissue was then incubated in three changes of propylene oxide for 10 min each, followed by infiltration of a hard resin with 30 min each in a 33%, and 66% resin diluted in propylene oxide, then in 100% resin overnight at room temperature after which it was flat-embedded with fresh resin and left to polymerize for 48 h at 60 °C. Flat-embedded tissue was then further embedded in a Beem capsule with fresh 100% resin and left to polymerize for a further 48 h at 60 °C. Thick, 500 nm resin sections counterstained with toluidine blue were collected for orientation and reference, and 80 nm ultrathin sections were collected on 200 hex copper grids (G200H-Cu) and contrasted with 2% aqueous uranyl acetate and lead citrate. To control against nonspecific staining, we used a staining protocol without incubation in primary antibodies. No immunogold particles were observed in those control sections (Supplementary Fig. 3).

### Image acquisition of chromogenic stained sections

Immunolabelled sections were imaged using the ‘Rescan Region of Interest (ROI)’ setting of the VSlide scanner (MetaSystems Hard & Software GmbH, Germany) running Metafer4 software (MetaSystems). A slide size of 76 × 24 mm was selected, and a pre-scan of the whole slide was generated using a 2.5x objective lens. The pre-scan image was automatically stitched together and saved into a ‘MetaClient’ directory.

#### Image acquisition of immunofluorescence-stained sections for 2D analysis

Images were collected using Zeiss ZEN 3.0 software on a Zeiss LSM710 inverted confocal microscope with a 0.63x objective lens and 0.6x zoom. For each of RgpB or Kgp stained sections, three replicate sections per case were imaged, and each section was sampled in three different positions along the substantia nigra pars compacta, which was delineated by the presence of neuromelanin (NM)-positive cells. Each position was imaged as a 2 × 2 tiled image stitched with 10% overlap, resulting in a sample area of 427.21 × 427.21 μm for each position. Two z-levels up to 5 μm apart were imaged for each position. NM is a dark pigment that is visible with transmitted light without the requirement for any immunohistochemical markers. It is not visible under fluorescence. Transmitted light was used to obtain “brightfield” images of NM, captured by a photomultiplier tube.

#### Image acquisition of immunofluorescence-stained sections for 3D rendering

Imaging was conducted on a Zeiss LSM 710 inverted confocal microscope using a 63x objective (NA 1.3). NM-positive nigral cells which also co-labeled with anti-αSynp and CAB102.1 were imaged in their entirety with a z-resolution of 0.32 μm. NM is a dark pigment that is visible with transmitted light without the requirement for any immunohistochemical markers. NM is not visible under fluorescence. Transmitted light was used to obtain “brightfield” images of NM, captured by a photomultiplier tube and was inverted and thresholded before rendering in 3D. The use of transmitted light for NM produced a 3D render that retained some detail from above and below the plane of focus, which gave rise to a marginal overestimation of the size of the NM mass parallel to the light path, i.e., along the z-axis. 3D renders using mixed mode rendering were produced in Zeiss ZEN 3.0.

### Image acquisition for TEM analysis

Sections were visualized with a Tecnai G2 Spirit Twin transmission electron microscope mounted with an Olympus-Soft Imaging Systems Morada digital camera.

### Image analysis and quantification of chromogenic images

To determine the load of CAB101 and CAB102.1 per nigral dopamine neuron we performed a semiquantitative analysis scoring the overlap of CAB101 and CAB102.1 with neuromelanin-containing neurons. A blinded investigator scored the level of overlap of either CAB101 or CAB102.1 with neuromelanin on a score from 0 (no overlap of cells) to 10 (complete overlap). Three sections were analyzed per case and the averaged used to generate the overall score per case. For the integrated intensity analysis, the same sections were imaged as described above and were analyzed using Metamorph software (Metamorph Offline v.7.8.0, Molecular Devices). Image analysis was conducted using the “Count Nuclei” algorithm and the integrated intensity measurements were logged.

### Image analysis and quantification of immunofluorescence images

Quantification was carried out using an automated image segmentation pipeline optimized in ImageJ 1.53 c. A total of five different markers were assessed. Hoechst staining was used as a nuclear marker for all cells. NeuN is typically expressed in neuronal cells and thus used as a neuronal cell marker. Not all neurons express neuromelanin, but in general, neuromelanin cells are neuronal and express NeuN, thus NeuN + NM combined was used as a marker for the neuronal cell population in the SNpc, whilst the absence of either was classed as non-neuronal. Masks for NeuN, NM, and Hoechst were used to determine neuronal and non-neuronal cell populations along with a Voronoi watershed (Supplementary Fig. 2). To outline the two staining patterns (diffuse and punctate) observed with RgpB and Kgp staining, two separate masks were generated for these markers by applying a size filter to the mask generated for RgpB and Kgp. Objects with an area less than 10 μm^2^ were classified as punctate, with the remainder classified as diffuse. In addition to the mask, to identify αSynp staining these diffuse and punctate masks were then used to determine the distribution and localization of αSynp and RgpB or Kgp in the total cell population, neuromelanin cell population, neuronal cell population, and the non-neuronal cell population (Supplementary Fig. 2).

### Lysine-gingipain digestion of recombinant α-synuclein

Recombinant α-synuclein (rαSyn; 200ug/mL; Anaspec, USA) was added to Kgp or RgpB (a kind gift of Barbara Potempa, University of Louisville) to 100 mM Tris, 75 mM NaCl, 2.5 mM CaCl2, 10 mM Cys-HCl pH 7.5 buffer at 37 °C. The reaction was stopped at the indicated times (0.5, 1, 2, 5 min) by adding 4x Laemmli buffer (BioRad, USA) with 10% 2-mercaptoethanol. Samples were heated to 95 °C for 10 min and loaded on an Any kD precast polyacrylamide gel (BioRad, USA). After gel electrophoresis gels immersed in Coomassie Blue Reagent (ThermoFisher, USA) for 1 h at room temperature followed by imaging using a BioRad GelDoc imaging system.

### Proteomics

Intact mass analysis was performed on full length and Kgp digested rαSyn to analyze intact proteins and fragments. Peptide analysis was performed using rαSyn digested with either trypsin or Kgp. Over 99% of the sequence could be mapped in the trypsin digested sample. Samples were prepared at a protein concentration of 0.5 mg/mL in 8 M urea and further diluted 1:5 with 10% acetonitrile and 0.1% formic acid. Liquid chromatography-mass spectroscopy (LCMS) analysis was performed with an injection volume of 10 µL. Chromatography was performed using a Dionex UltiMate ® 3000 FLM HPLC, in tandem with a QExactive TM Orbitrap Mass Spectrometer. For Intact mass analysis, a 20-min gradient was performed using an Agilent TM PLRP S 1000 A 5 µm column. For Peptide analysis, a 1-h gradient was performed using a Waters TM Acquity BEH C18 1.7 µm, 2.1 × 50 mm column. Data analysis was accomplished using Thermo Xcalibur Qualbrowser TM, PEAKS®.

### Statistics

For chromogenic quantification the results displayed as the median +/− the interquartile range. Significance was tested with the Mann−Whitney test. For immunofluorescence quantification results are displayed as the mean +/− the standard deviation, and significance was tested for using an unpaired t-test with Welch’s correction or ANOVA with Tukey’s multiple comparison test. All statistics and graphs were performed and created with GraphPad Prism 10 (Dotmatics, Boston, MA).

### Supplementary information

Supplementary Figures

Reporting-Summary

## Data Availability

The datasets used and/or analyzed during the current study are available from the corresponding authors on request.
