# Supplementary material for: Ultrastructural localization of Porphyromonas gingivalis gingipains in the substantia nigra of Parkinson’s disease brains
Source: NPJ Parkinsons Dis. 2024 Apr 25;10:90. doi: 10.1038/s41531-024-00705-2 (PMC11045759; doi:10.1038/s41531-024-00705-2)
Supplement: Supplementary file 1 — Supplementary Figures [file 41531_2024_705_MOESM1_ESM.pdf]

Supplemental Figures

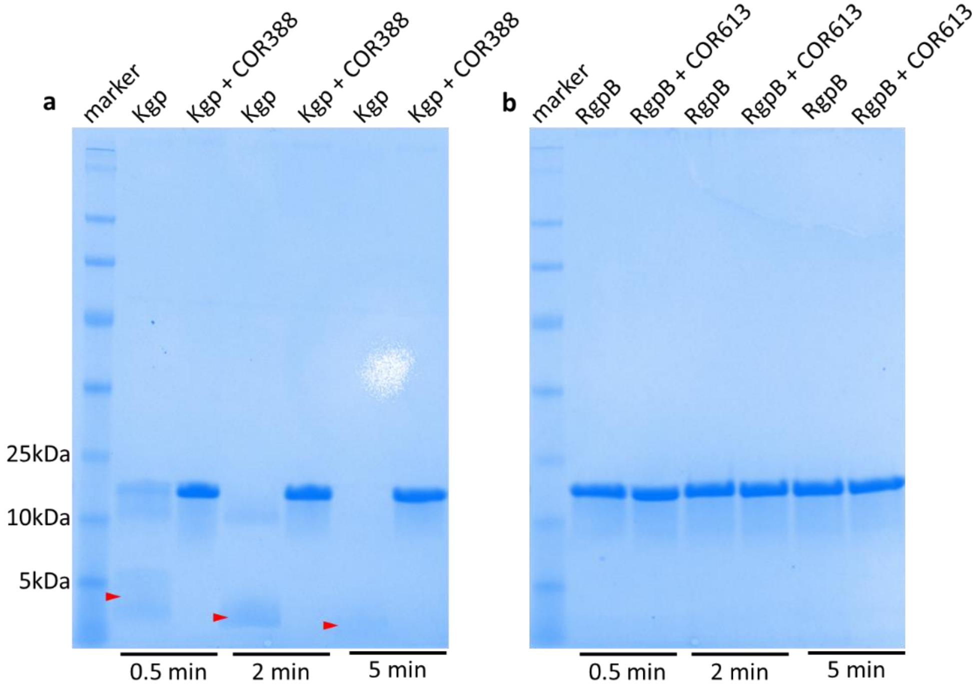

Supplemental Figure 1: Coomassie gels of rαSyn exposed to Kgp or RgpB. **a:** Coomassie gel of recombinant αSyn exposed to 30 sec, 2 min or 5 min of Kgp, with or without Kgp inhibitor COR388. Fragmented αSyn appears as a faint diffuse band at 5 kDa and below after 30 seconds of Kgp digestion and appears fainter with a wider spread at 2 min and 5 min digestion (red arrowheads). **b:** Coomassie gel of recombinant αSyn exposed to 30 sec, 2 min or 5 min of RgpB, with or without RgpB inhibitor COR613. Fragmented αSyn appears as a faint diffuse band at 5 kDa and below after 30 seconds of Kgp digestion and appears fainter with a wider spread at 2 min and 5 min digestion (red arrowheads). Enzymatic potency of both Kgp and RgpB was confirmed with a fluorogenic assay using the substrates Z-His-Glu-Lys-MCA and Boc-Phe-Ser-Arg-MCA for Kgp and RgpB respectively, as described in Dominy et al (2019).

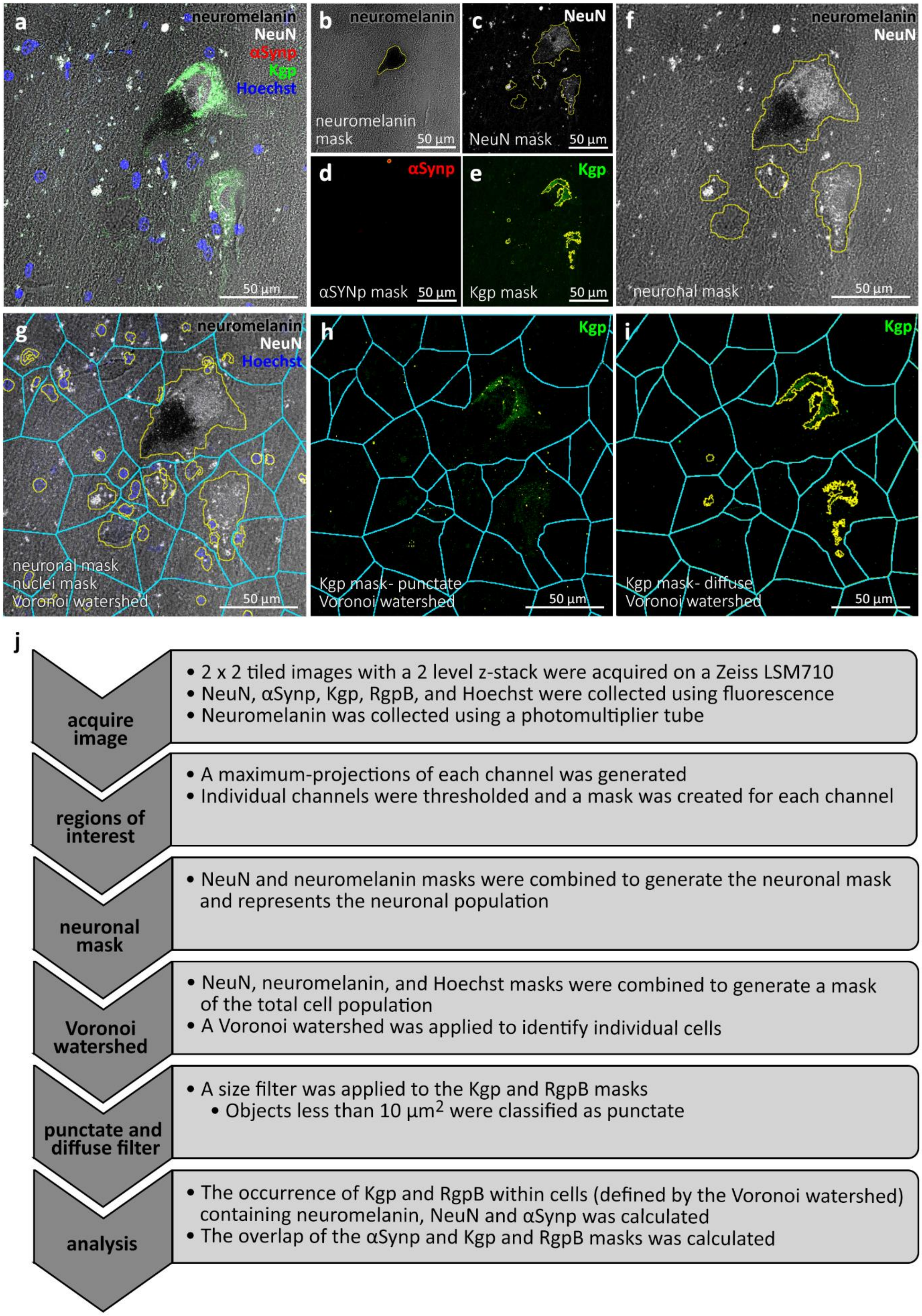
a
neuromelanin
NeuN
αSynp
Kgp
Hoechst
50 µm
b
neuromelanin
neuromelanin mask
50 µm
c
NeuN
NeuN mask
50 µm
d
αSynp
αSYNp mask
50 µm
e
Kgp
Kgp mask
50 µm
f
neuromelanin
NeuN
neuronal mask
50 µm
g
neuromelanin
NeuN
Hoechst
neuronal mask
nuclei mask
Voronoi watershed
50 µm
h
Kgp
Kgp mask- punctate
Voronoi watershed
50 µm
i
Kgp
Kgp mask- diffuse
Voronoi watershed
50 µm
j
acquire image
• 2 x 2 tiled images with a 2 level z-stack were acquired on a Zeiss LSM710
• NeuN, αSynp, Kgp, RgpB, and Hoechst were collected using fluorescence
• Neuromelanin was collected using a photomultiplier tube
regions of interest
• A maximum-projections of each channel was generated
• Individual channels were thresholded and a mask was created for each channel
neuronal mask
• NeuN and neuromelanin masks were combined to generate the neuronal mask and represents the neuronal population
Voronoi watershed
• NeuN, neuromelanin, and Hoechst masks were combined to generate a mask of the total cell population
• A Voronoi watershed was applied to identify individual cells
punctate and diffuse filter
• A size filter was applied to the Kgp and RgpB masks
• Objects less than 10 µm2 were classified as punctate
analysis
• The occurrence of Kgp and RgpB within cells (defined by the Voronoi watershed) containing neuromelanin, NeuN and αSynp was calculated
• The overlap of the αSynp and Kgp and RgpB masks was calculated

Supplemental figure 2 (previous page): Formalin fixed paraffin embedded immunofluorescence analysis pipeline. **a-i:** Kgp co-labeling using the CAB102.1 antibody (green) with αSynp (red), NeuN (white), Hoechst (blue), and endogenous neuromelanin (black) in the PD SNpc. **a:** Merge of all 5 channels. **b:** neuromelanin (black) captured by a photomultiplier tube and the generated mask outline is displayed in yellow. **c:** NeuN (white) and the generated mask outline is displayed in yellow. **d:** αSynp (red) and the generated mask outline is displayed in yellow. **e:** Kgp detected with the CAB102.1 antibody (green) and the generated mask outline is displayed in yellow. **f:** Masks generated from neuromelanin (black) and NeuN (white) were combined to form the neuronal mask. **g:** The neuronal mask (yellow) and the mask for nuclei generated using Hoechst labelling (yellow) were combined to represent the total cellular population and was then segmented with a Voronoi watershed (cyan). **h:** Kgp detected with the CAB102.1 antibody (green) and the outline for the generated mask of punctate signal is displayed in yellow and segmented with the Voronoi mask (cyan). **i:** Kgp detected with the CAB102.1 antibody (green) and the outline for the generated mask of diffuse signal is displayed in yellow and segmented with the Voronoi mask (cyan). **j:** Flow diagram summary of a-i.

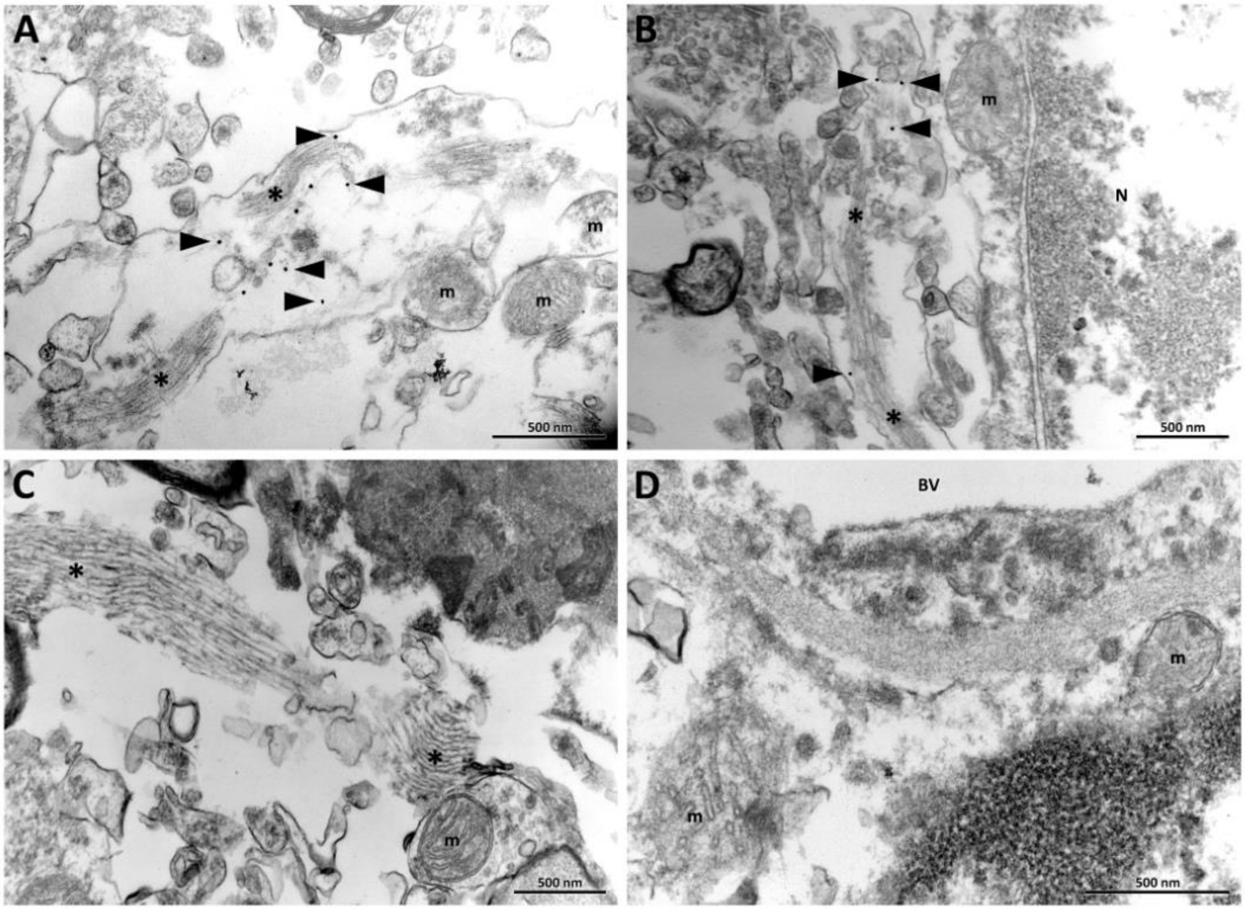

Supplemental Figure 3: positive and no primary controls for immunogold staining. **A/B:** Glial Fibrillary Acid Protein (GFAP) immunogold staining was used as a positive control. 15 nm gold particles (black arrowhead) are observed closely associated with filament structures (v). No gold particles were observed inside mitochondria (m) or nuclei (N). These ultrathin sections were collected 500nm (A) or 5 μm (B) from the surface of the 50 μm flat embedded section. **C/D:** To ensure that we are not observing non-specific binding of gold particles to the tissue we included a no primary control. Both the 6 nm and 15 nm gold secondaries were used while primary antibody was omitted from the IHC procedure to obtain a no primary negative control. Without primary antibody staining, we can determine if there is any non-specific gold particle binding that may be occurring in these experiments. After careful observation, we were unable to detect any gold staining. Asterisk: filament structure; m: mitochondria; BV: blood vessel. Scale bars: 500 nm.

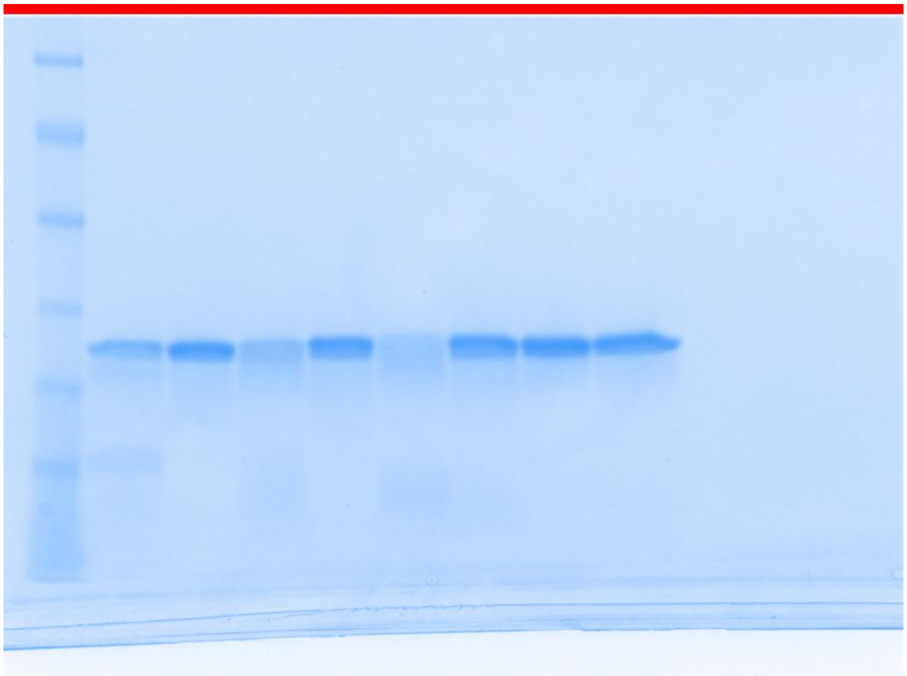

Supplemental Figure 4: Uncropped Coomassie gel of the data presented in figure 2a.

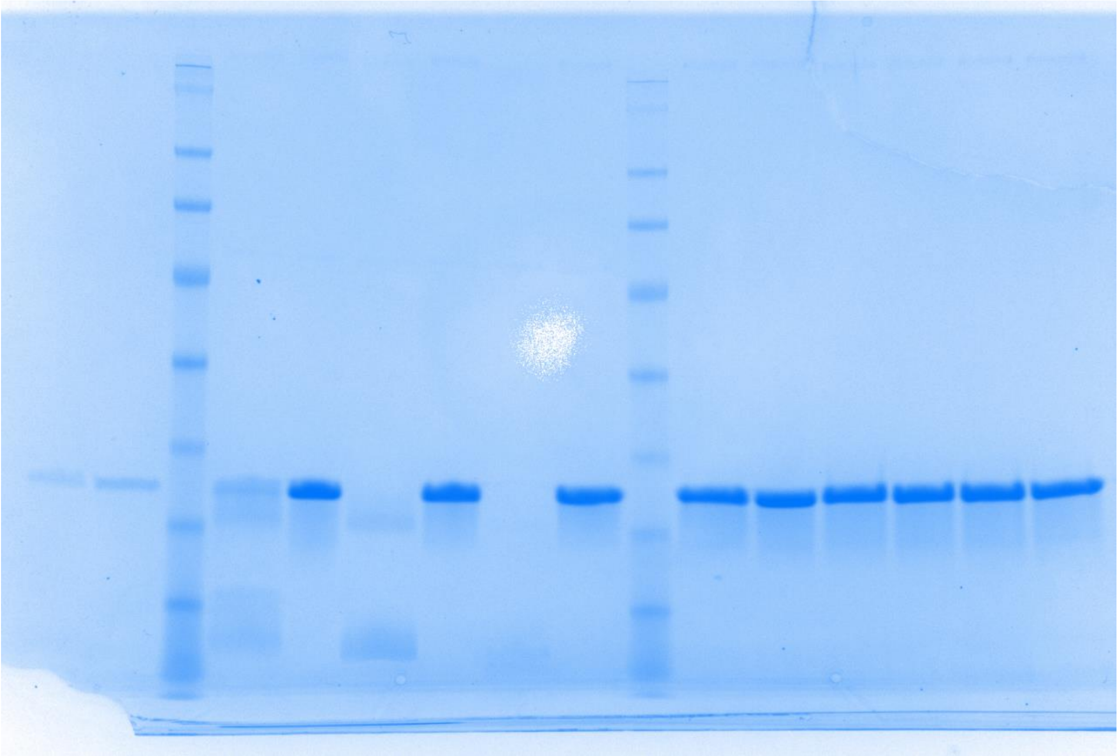

Supplemental Figure 5: Uncropped Coomassie gels of the data presented in supplemental figure 1.
