# Supplementary material for: Ultrastructural localization of Porphyromonas gingivalis gingipains in the substantia nigra of Parkinson’s disease brains
Source: NPJ Parkinsons Dis. 2024 Apr 25;10:90. doi: 10.1038/s41531-024-00705-2 (PMC11045759; doi:10.1038/s41531-024-00705-2)
Supplement: Supplementary file 2 — Reporting-Summary [file 41531_2024_705_MOESM2_ESM.pdf]

nature portfolio

Corresponding author(s): Dr Florian Ermini
NPJPARKD-02341R

Last updated by author(s): Jan 15, 2024

Reporting Summary

Nature Portfolio wishes to improve the reproducibility of the work that we publish. This form provides structure for consistency and transparency in reporting. For further information on Nature Portfolio policies, see our Editorial Policies and the Editorial Policy Checklist.

Statistics

For all statistical analyses, confirm that the following items are present in the figure legend, table legend, main text, or Methods section.

n/a	Confirmed	
☐	☒	The exact sample size (n) for each experimental group/condition, given as a discrete number and unit of measurement
☐	☒	A statement on whether measurements were taken from distinct samples or whether the same sample was measured repeatedly
☐	☒	The statistical test(s) used AND whether they are one- or two-sided<br>*Only common tests should be described solely by name; describe more complex techniques in the Methods section.*
☒	☐	A description of all covariates tested
☐	☒	A description of any assumptions or corrections, such as tests of normality and adjustment for multiple comparisons
☐	☒	A full description of the statistical parameters including central tendency (e.g. means) or other basic estimates (e.g. regression coefficient) AND variation (e.g. standard deviation) or associated estimates of uncertainty (e.g. confidence intervals)
☐	☒	For null hypothesis testing, the test statistic (e.g. F, t, r) with confidence intervals, effect sizes, degrees of freedom and P value noted<br>*Give P values as exact values whenever suitable.*
☒	☐	For Bayesian analysis, information on the choice of priors and Markov chain Monte Carlo settings
☒	☐	For hierarchical and complex designs, identification of the appropriate level for tests and full reporting of outcomes
☒	☐	Estimates of effect sizes (e.g. Cohen's d, Pearson's r), indicating how they were calculated

Our web collection on statistics for biologists contains articles on many of the points above.

Software and code

Policy information about availability of computer code

Data collection	Metafer4 software (MetaSystems Hard & Software GmbH, Germany), Zeiss ZEN 3.0, ImageJ 1.53c, Thermo Xcalibur Qualbrowser TM, PEAKS®
Data analysis	GraphPad Prism

For manuscripts utilizing custom algorithms or software that are central to the research but not yet described in published literature, software must be made available to editors and reviewers. We strongly encourage code deposition in a community repository (e.g. GitHub). See the Nature Portfolio guidelines for submitting code & software for further information.

Data

Policy information about availability of data

All manuscripts must include a data availability statement. This statement should provide the following information, where applicable:

- Accession codes, unique identifiers, or web links for publicly available datasets
- A description of any restrictions on data availability
- For clinical datasets or third party data, please ensure that the statement adheres to our policy

The datasets used and/or analyzed during the current study are available from the corresponding authors on reasonable request.

Research involving human participants, their data, or biological material

Policy information about studies with human participants or human data. See also policy information about sex, gender (identity/presentation), and sexual orientation and race, ethnicity and racism.

Reporting on sex and gender	Age and sex of cases included in the study is reported in the methods section. Cases were age and sex matched with an average age of 79.7 +/- 7.2 and 78.3 +/- 9.8 (P<0.89) years in normal and PD respectively.
Reporting on race, ethnicity, or other socially relevant groupings	*Please specify the socially constructed or socially relevant categorization variable(s) used in your manuscript and explain why they were used. Please note that such variables should not be used as proxies for other socially constructed/relevant variables (for example, race or ethnicity should not be used as a proxy for socioeconomic status).*<br>*Provide clear definitions of the relevant terms used, how they were provided (by the participants/respondents, the researchers, or third parties), and the method(s) used to classify people into the different categories (e.g. self-report, census or administrative data, social media data, etc.)*<br>*Please provide details about how you controlled for confounding variables in your analyses.*
Population characteristics	Characteristics included are: neurological diagnosis, age, sex, cause of death and pathological diagnosis
Recruitment	The human tissue was donated to the Brain Bank with consent from the donors' families.
Ethics oversight	This project was approved by the Health and Disability Ethics Committee of Neurological Foundation Human Brain Bank at the University of Auckland, Centre for Brain Research

Note that full information on the approval of the study protocol must also be provided in the manuscript.

Field-specific reporting

Please select the one below that is the best fit for your research. If you are not sure, read the appropriate sections before making your selection.

☒ Life sciences ☐ Behavioural & social sciences ☐ Ecological, evolutionary & environmental sciences

For a reference copy of the document with all sections, see nature.com/documents/nr-reporting-summary-flat.pdf

Life sciences study design

All studies must disclose on these points even when the disclosure is negative.

Sample size	Sample size was restricted by the availability of samples. The limitations of the sample size a discusses in the discussion section of the manuscript.
Data exclusions	no data was excluded.
Replication	Three replicate sections per case were imaged, and each section was sampled in three different positions along the substantia nigra pars compacta.
Randomization	Cases were age and sex matched with an average age of 79.7 +/- 7.2 and 78.3 +/- 9.8 (P<0.89) years in normal and PD respectively.
Blinding	The analysis was performed by a blinded investigator.

Reporting for specific materials, systems and methods

We require information from authors about some types of materials, experimental systems and methods used in many studies. Here, indicate whether each material, system or method listed is relevant to your study. If you are not sure if a list item applies to your research, read the appropriate section before selecting a response.

Materials & experimental systems

n/a	Involved in the study	
☐	☒	Antibodies
☒	☐	Eukaryotic cell lines
☒	☐	Palaeontology and archaeology
☒	☐	Animals and other organisms
☒	☐	Clinical data
☒	☐	Dual use research of concern
☒	☐	Plants

Methods

n/a	Involved in the study	
☒	☐	ChIP-seq
☒	☐	Flow cytometry
☒	☐	MRI-based neuroimaging

Antibodies

Antibodies used

CAB101 RgpB, Rabbit (0.5 mg/ml) Cortexyme, Inc.
CAB102.1 Kgp, Rabbit (0.46 mg/ml) Cortexyme
Anti-Alpha-synuclein (phosphoS129) antibody [P-syn/81A], Mouse monoclonal Abcam Cat# ab184674, RRID:AB_2819037)
Anti-NeuN purified, Guinea Pig polyclonal Millipore Cat# ABN90P, RRID:AB_2341095
Goat anti-Rabbit IgG (H+L) Highly Cross-Adsorbed Secondary Antibody, Alexa Fluor 488 Thermo Fisher Scientific Cat# A-11034, RRID:AB_2576217
Goat anti-Mouse IgG (H+L) Highly Cross-Adsorbed Secondary Antibody, Alexa Fluor 594 Thermo Fisher Scientific Cat# A-11032, RRID:AB_2534091
Goat Anti-Guinea Pig IgG (H+L) Highly Cross-adsorbed Antibody, Alexa Fluor 647 Conjugated Molecular Probes Cat# A-21450, RRID:AB_141882
Goat anti-Rabbit IgG (H+L) coupled with 6 nm gold, EM grade Electron Microscopy Science Auron 106.011 Cat# 25103 TEM; 1:50
Goat anti-Mouse IgG (H+L) coupled with 15 nm gold, EM grade Electron Microscopy Science Auron 115.022 Cat# 25132 TEM; 1:50

Validation

CAB101 and CAB102.1 have been validated by evaluation of dilution series, staining of positive controls, negative staining after absorption with immunogen and no-primary controls and negative staining of negative controls.
Anti-Alpha-synuclein (phosphoS129) antibody [P-syn/81A], was tested by manufacturer by IHC on positive control and sites 24 references on their product website.
Anti-NeuN purified, Guinea Pig polyclonal Millipore Cat# ABN90P was evaluated by manufacturer by Western Blot in mouse E16 brain tissue lysate and sites 74 references on their product website.

Plants

Seed stocks

Report on the source of all seed stocks or other plant material used. If applicable, state the seed stock centre and catalogue number. If plant specimens were collected from the field, describe the collection location, date and sampling procedures.

Novel plant genotypes

Describe the methods by which all novel plant genotypes were produced. This includes those generated by transgenic approaches, gene editing, chemical/radiation-based mutagenesis and hybridization. For transgenic lines, describe the transformation method, the number of independent lines analyzed and the generation upon which experiments were performed. For gene-edited lines, describe the editor used, the endogenous sequence targeted for editing, the targeting guide RNA sequence (if applicable) and how the editor was applied.

Authentication

Describe any authentication procedures for each seed stock used or novel genotype generated. Describe any experiments used to assess the effect of a mutation and, where applicable, how potential secondary effects (e.g. second site T-DNA insertions, mosiacism, off-target gene editing) were examined.
